# COSMO-RS guided screening of deep eutectic solvent extraction for essential oil from *Ligusticum chuanxiong*: optimization, composition analysis, and acetylcholinesterase inhibitory activity

**DOI:** 10.1039/d6ra04452b

**Published:** 2026-09-01

**Authors:** Zhouwei Hong, Liu Tang, Tong Xia, Gang Liu, Yuanguo Xiong, Lian Liu, Xu Cai

**Affiliations:** a Pharmaceutical Department, Renmin Hospital of Wuhan University Wuhan Hubei 430060 China 2021caixu@whu.edu.cn; b Department of Anesthesiology, Renmin Hospital of Wuhan Universityy Wuhan Hubei 430060 China elysiall@whu.edu.cn; c College of Pharmacy, Hubei University of Chinese Medicine Wuhan Hubei 430065 China 13638697959@163.com

## Abstract

Essential oil of *Ligusticum chuanxiong* (EO-LC) exhibits notable diverse biological activities, presenting considerable potential for aromatherapy and pharmaceutical applications. Deep eutectic solvents (DESs) are an effective extraction solvent for essential oils. According to thermodynamic analysis from the COSMO-RS model and subsequent experimental validation, betaine/lactic acid (2 : 1) was identified as the optimum candidate and the optimal extraction conditions were established as follows: a water content of 48%, a liquid-to-material ratio of 8.5 : 1 mL g^−1^, and an extraction time of 2.9 h. Compared to traditional steam distillation (0.44%), DES-based extraction significantly increased the EO-LC yield to 1.14 ± 0.07%, while simultaneously improving the chemical diversity and the relative contents of its major active constituents. Furthermore, EO-LC displayed excellent acetylcholinesterase inhibitory activity (IC_50_ value of 41.25 µg mL^−1^) through hydrogen bonding and hydrophobic interactions and van der Waals forces were identified as the primary driving forces for this efficient extraction of EO-LC. This work provides robust theoretical support for the rational design of DESs in EO-LC extraction, and offers novel insights into the efficient utilization of EO-LC.

## Abstract 2

A rational design strategy of DES was explored for extracting essential oil from *Ligusticum chuanxiong* (EO-LC) and an efficient DES extraction method was established, by which the chemical composition and bioactivity of EO-LC were improved.

## Graphical abstract

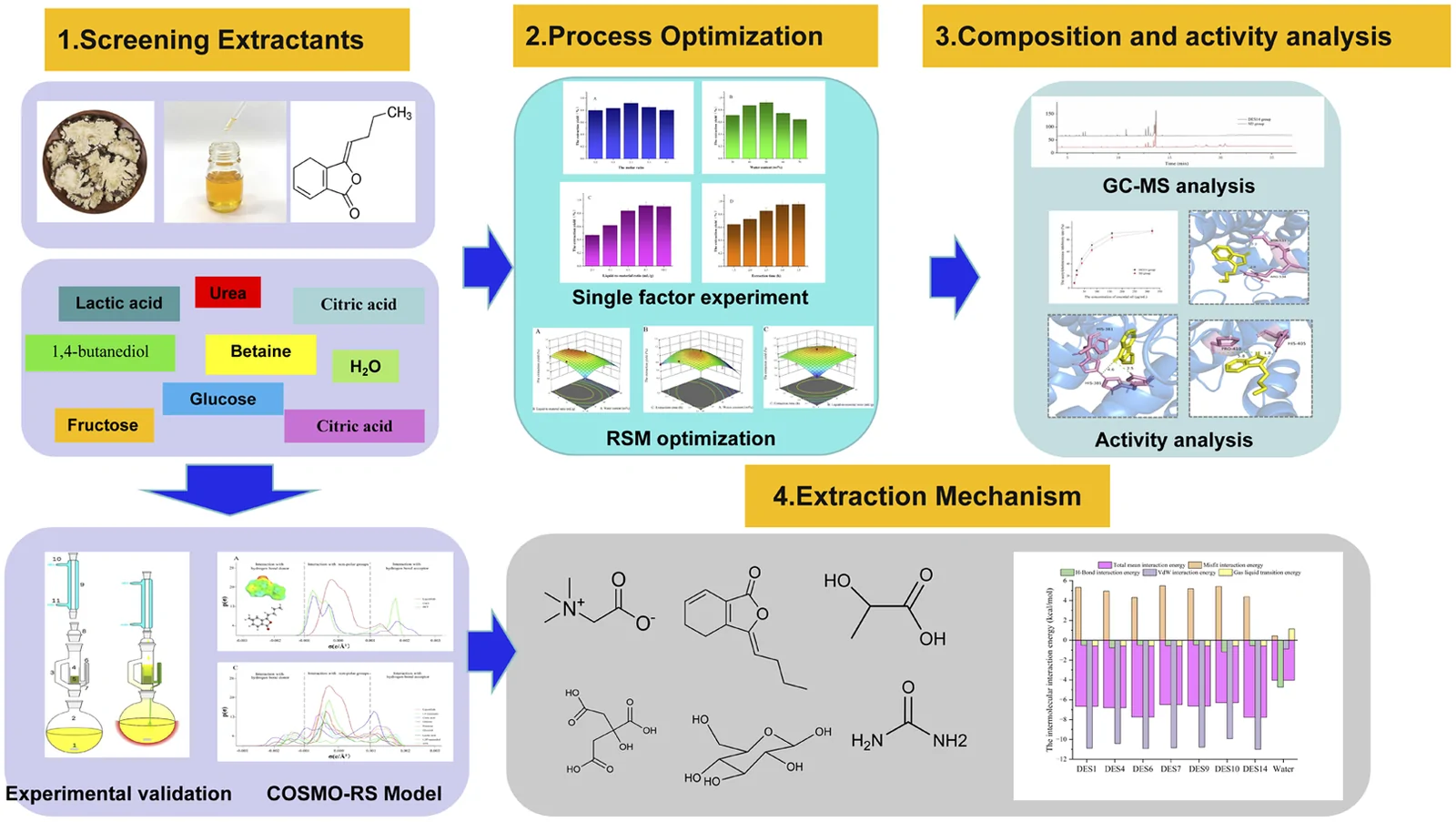

## 1 Introduction

*Ligusticum chuanxiong* Hort., derived from the genus Ligusticum in the family Apiaceae, is commonly employed to alleviate headaches, rheumatic arthralgia, and irregular menstruation.^1^ Owing to its exclusive herbal aroma, essential oil of *Ligusticum chuanxiong* (EO-LC) has been widely utilized as an additive of health foods and cosmetics. As a critical bioactive fraction, EO-LC is also the core constituent in several traditional Chinese medicine patent preparations, such as Chuanxiong chatiao powder, Naoxintong capsule, and Zhuifeng Tougu pill. GC-MS analysis has identified a diverse variety of components in EO-LC, including ligustilide, butylidenephthalide, senkyunolide, and terpinen-4-ol.^2^ Ligustilide (LIG) is the predominant active component in EO-LC, comprising more than 50% of its total composition. LIG serves not only as a key quality control marker but also as the principal active constituent responsible for the pharmacological activities of EO-LC.^3^ Numerous studies have confirmed that EO-LC possesses a wide range of biological activities, such as anti-inflammation, neuroprotection and anticancer activity.^4^ For instance, EO-LC could remarkably reduce the overexpression of inflammatory mediators and pro-inflammatory cytokines through inhibiting the NF-κB pathway, thereby ameliorating neuroinflammation.^5^ Following intranasal administration, EO-LC demonstrated markedly superior efficacy in ameliorating neurological deficits and cerebral pathological injury.^6^ In addition, EO-LC was capable of promoting the penetration of temozolomide into glioma cells and enhancing its anti-tumor efficacy.^7^ Collectively, these results demonstrated the distinctive pharmacodynamic profiles of EO-LC, and underscored its great potential for developing aromatic therapeutics.

Various extraction techniques have been developed and extensively employed for the extraction of essential oil (EO), including traditional steam distillation (SD), organic solvent extraction, and supercritical CO_2_ extraction. Nevertheless, the application of these extraction methods is often limited by inefficient cell wall disruption, prolonged thermal exposure, potential contamination risks from residual organic solvents, and high equipment investment costs.^8^ Deep eutectic solvents (DESs), a promising green alternative, are eutectic mixtures formed through robust hydrogen-bond (H-bond) interaction networks between H-bond donors (HBDs) and H-bond acceptors (HBAs).^9^ Due to the distinctive interaction behaviors, DESs exhibit excellent solubilization capacity, which have attracted considerable research attention in the food and pharmaceutical fields.^10^ Consequently, DESs possess robust solubilization capacity for lignin, polysaccharides, and pectin, have emerged as a promising candidate medium for EO extraction. For instance, ChCl and citric acid (1 : 0.9 of molar ratio) were employed as the extraction solvent, which significantly increased the yield of *Litsea cubeba* from 3.20% to 4.70%.^11^ And ChCl and lactic acid were selected for enriching agarwood EO.^12^ ChCl and lactic acid (1 : 3 of molar ratio) with 40 wt% water were determined to be the optimum DES for extracting EO from *Cuminum cyminum* L.^13^ In comparison with the traditional SD technique, EO extracted by DES have not only achieved superior extraction efficiencies but also exhibited distinct and robust antioxidant, antibacterial, and enzyme inhibitory activities.^11–13^ Moreover, EO extracted using DES often contain a diverse array of distinctive compounds that are typically not detected in EO derived by SD method. Hence, the selection of suitable DESs is essential for achieving the efficient extraction of EO-LC and facilitating its subsequent practical applications.

Owing to the structural complexity and compositional diversity of DESs, traditional experimental solvent screening is plagued by long experimental cycles and labor-intensive operations. These shortcomings greatly diminish the screening efficiency for DESs.^14^ With the rapid development of computational chemistry theories, numerous computational models have been well established. Conductor-like screening model for real solvents (COSMO-RS), a theoretical model rooted in statistical thermodynamics, is extensively employed for molecular structure optimization, reaction mechanism exploration, and the simulation of material properties.^15^ By importing theoretical chemical structure data into the COSMO-RS model, key parameters can be efficiently computed, such as *σ*-profiles, solubility, and intermolecular forces.^16–18^ This high-throughput prediction approach enables rapid screening of DESs with high-efficiency extraction potential. According to the activity coefficient at infinite dilution, benzoic acid and fenchyl alcohol were constructed for the effective extraction of artemisinin from *Artemisia annua* L. leaves.^16^ According to the relative solubility, betaine and maleic acid were identified as the optimal DESs for extracting polyphenol from *Moringa oleifera* Lam.^17^ To recover coumarin from *Pterocaulon polystachyum*, DES consisting of menthol and lauric acid was chosen as the optimal candidate.^18^ Therefore, the COSMO-RS model is a robust and computationally efficient theoretical framework for the accurate identification of optimal HBA and HBD pairs in the selective extraction of target compound. Despite the considerable advantages of COSMO-RS model in DESs screening, the application of COSMO-RS model to guide EO extraction has rarely been reported.

Herein, the COSMO-RS model combined with experimental extraction was adopted to screen the optimal DES for the extraction of EO-LC. On this basis, single-factor experiments and response surface methodology (RSM) were integrated to optimize DES-assisted extraction parameters for EO-LC. The technical advantages of DES extraction are compared by analyzing chemical composition and the relative contents. Additionally, acetylcholinesterase (AChE) inhibitory activity and molecular docking were systematically performed to clarify the potential for developing aromatic therapeutics from EO-LC. Finally, the underlying extraction mechanism of DES was further elucidated *via* COSMO-RS. This work not only lays a solid theoretical basis for the targeted design of DESs for EO extraction, but also offers new perspectives on the high-efficiency extraction and value utilization of EO-LC.

## 2 Materials and methods

### 2.1 Chemicals and equipments

#### 2.1.1 Chemicals

*Ligusticum chuanxiong* Hort. was purchased from PKU HealthCare Corp., Ltd (Chongqing, China). Choline chloride (ChCl, C_5_H_14_ClNO, AR, ≥99%), betaine (BET, C_5_H_11_NO_2_, ≥99%), lactic acid (LA, C_3_H_6_O_3_, ≥99%), citric acid (CA, C_6_H_8_O_7_, AR, ≥99%), 1,4-butanediol (BUT, C_4_HO_2_, AR, ≥99%) and 1,3-propanediol (PDO, C_3_H_8_O_2_, AR, ≥99%) were obtained from Shanghai Aladdin Biochemical Technology Co.,Ltd (Shanghai, China). Acetylcholinesterase (AChE), 5,5′-dithiobis(2-nitrobenzoic acid) (DTNB), acetylthiocholine iodide (ATChI) were derived from Yuanye Biotechnology Co., Ltd (Shanghai, China). Phosphate buffer saline (PBS) and *n*-hexane (C_6_H_14_, ≥99%) were obtained from Shanghai Titan Scientific Co., Ltd (Shanghai, China).

#### 2.1.2 Equipments

HH-J1 thermostat water bath (Changzhou Jebsen Instrument Co., Ltd, China), SpectraMax iD3 microplate reader (Molecular Devices Co., Ltd, USA), GCMS-QP2010 Ultra (Shimadzu Co., Japan).

### 2.2 Thermodynamic analysis

As the principal active constituent of EO-LC, ligustilide (LIG) was employed as the target compound to facilitate the screening of optimal DESs. And HBAs and HBDs were retrieved from the PubChem database, and illustrated in Table S1. The candidate DESs were as depicted in Table S2. In accordance with the established calculation procedure of COSMO-RS model, geometry optimization was conducted using TURBOMOLE software at the BP86/def-TZVP level of theory. Subsequently, the COSMO files were imported into COSMOthermX software to analyze the thermodynamic characteristics (the *σ*-profile, *σ*-potential, and the activity coefficients of LIG) at 100 °C, which enabled the preliminary screening of suitable HBA and HBD candidates. Meanwhile, the intermolecular interaction energy was used to explore the extraction mechanism of EO-LC using DESs.

### 2.3 Experimental method

#### 2.3.1 Preparation of the candidate deep eutectic solvents

To synthesize the candidate DESs, specific combinations of LA, BUT, FRU, PDO, and CA were respectively mixed with ChCl and BET. The detailed molar ratios were summarized in Table S2. Then, the formulated mixtures were heated to 80 °C in a constant-temperature water bath (Tuohe Electromechanical Technology (Shanghai) Co., Ltd, Zhejiang, China) under continuous agitation, with an appropriate amount of water added. Once the solid components formed a transparent, homogeneous liquid, the successfully prepared DESs were transferred into airtight vials and preserved for subsequent experimental applications.

#### 2.3.2 Extraction process of essential oil from *Ligusticum chuanxiong*

The dried powder of *L. chuanxiong* (50 g) was homogenized with DESs or water at a liquid-to-material ratio of 8 : 1 mL g^−1^. After that, the mixture was soaked for 0.5 hours, then extracted by a micro-distillation apparatus under micro-boiling conditions for 3.0 hours. An appropriate amount of water was added during the extraction. Finally, the resulting oil–water mixture was extracted with *n*-hexane. After phase separation, the organic fraction was concentrated through solvent evaporation to obtain the EO-LC sample. The weight of EO-LC sample (*M*_1_, g) was recorded, and the yield of EO-LC (*Y*_1_, %) was determined *via*eqn (1):1
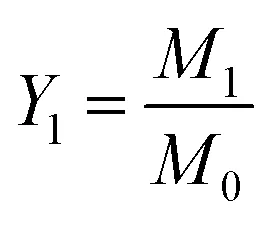
where *M*_0_, *M*_1_ referred to the weight of *L. chuanxiong* powder (*M*_0_ = 50 g), EO-LC sample, respectively.

#### 2.3.3 Optimization the extraction process on the extraction yield

According to the predefined procedure, the initial univariate experiments were conducted. As outlined in Table S3, the extraction parameters were investigated, including the molar ratio, water content (wt%), liquid-to-material ratio (mL g^−1^), and extraction time (h). Based on the outcomes of these preliminary experiments, a three-level, three-factor Box–Behnken design (BBD) was adopted for the extraction parameter optimization of EO-LC. Furthermore, this design enabled an in-depth analysis of synergistic and interactive relationships among the experimental variables. The coded levels were established *via* Design-Expert 13, and the practical RSM experimental conditions were summarized in Table S4.

### 2.4 Composition analysis of essential oil from *Ligusticum chuanxiong*

100 µL of EO-LC was dissolved in *n*-hexane, and diluted to a final volume of 1 mL. The resulting solution of EO-LC was then injected in split mode for the qualitative analysis of the chemical composition of EO-LC. The detailed GC-MS analysis conditions were described as follows: chromatographic conditions: column: SHIMADZU SH Rt™-U-Bond capillary column (30 m × 0.25 mm, 0.25 µm film thickness); carrier gas: high purity He gas; flow rate: 1.0 mL min; split ratio: 50 : 1; injection volume: 1 µL; injector temperature: 280 °C; temperature program: initial temperature was set at 60 °C and held for 2 min, ramped up to 180 °C at 10 °C min^−1^ and held for 1 min, then further increased to 280 °C at 10 °C min^−1^ and maintained for 5 min, with a total run time of 30.0 min. Mass spectrometry conditions: ionization source: EI source; electron energy: 70 eV; ion source temperature: 230 °C; transfer line temperature: 280 °C; quadrupole temperature: 150 °C; mass scan range: 50–500 *m*/*z*.

### 2.5 Analysis of AChE inhibitory activity and molecular docking

#### 2.5.1 Analysis of AChE inhibitory activity

The acetylcholinesterase inhibitory activity assay was adapted from the reported method,^19^ with minor modifications made to the original protocol. Briefly, 140 µL of PBS buffer, 10 µL of AChE solution (1000 U mL^−1^), and 20 µL of EO solution diluted in PBS buffer at different concentrations were sequentially added to a microplate. The reaction mixture was incubated at 37 °C for 60 min. Subsequently, 20 µL of DTNB solution and 10 µL of ATCI solution were added, and the reaction mixture was re-incubated under the same conditions. Finally, the absorbance of the mixed reaction solution was determined with a microplate reader at 412 nm. The AChE inhibition rate (*Y*_2_, %) was subsequently calculated *via*eqn (2):2
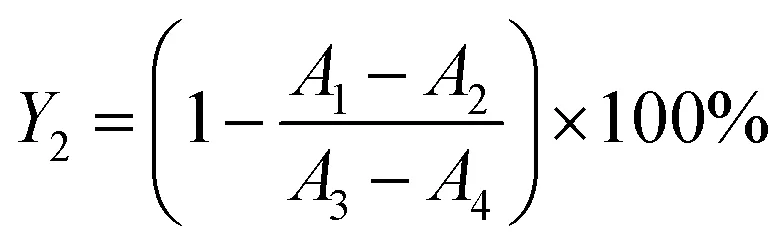
where *A*_1_, *A*_2_, *A*_3_, *A*_4_: absorbance of the reaction mixture, the reaction mixture without AChE, the blank reaction mixture without sample, the reaction mixture without AChE and EO-LC.

#### 2.5.2 Molecular docking

To elucidate the molecular mechanism underlying the AChE inhibitory activity of EO-LC, the key components with a threshold >2% were selected. The 3D structures of the candidate active ingredients were retrieved from the PubChem database (https://pubchem.ncbi.nlm.nih.gov/) and converted to the mol2 format using ChemDraw software. The protein structure of AChE (ID: 7E3H) was downloaded from the RCSB PDB database, followed by the removal of water molecules and non-protein small molecules *via* PyMOL software. The center parameters of AChE were set as *X* = −47.646, *Y* = 20.681, *Z* = −55.523. Subsequently, molecular docking between ligand and AChE was carried out *via* AutoDock Vina to acquire their corresponding binding energies. Finally, the binding sites and hydrogen bond interactions of ligand–receptor complexes were visualized and analyzed by PyMOL software.

## 3 Results and discussion

### 3.1 Thermodynamic analysis of ligustilide

**Fig. 1:**
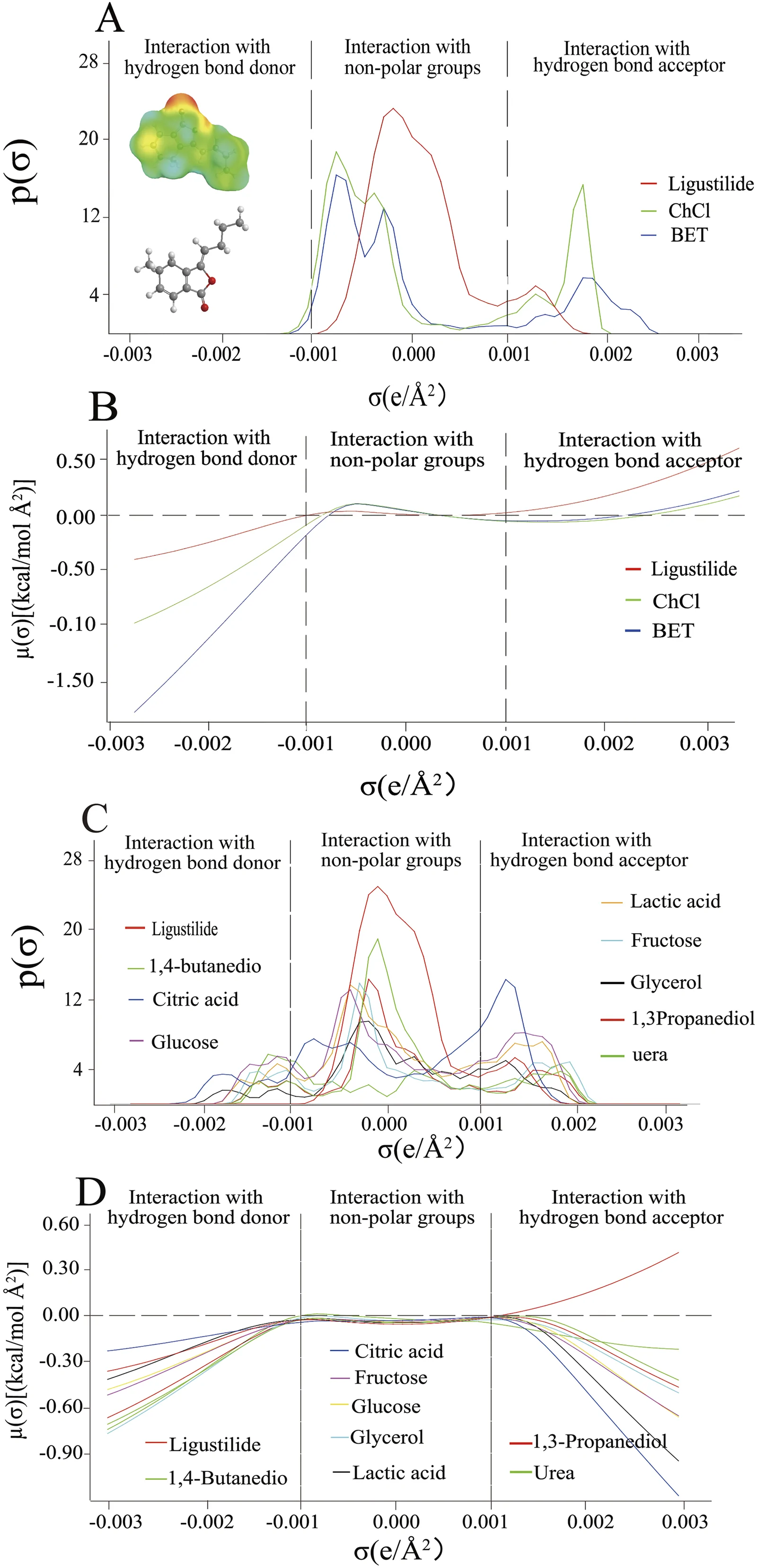
(A) The *σ*-profile of ligustilide and hydrogen bond acceptors. (B) The *σ*-potential of ligustilide and hydrogen bond acceptors; (C) the *σ*-profile of ligustilide and hydrogen bond donors. (D) The *σ*-potential of ligustilide and hydrogen bond donors.

The efficiency of the extraction process is inherently governed by the physicochemical properties of solvent system coupled with tunable operational parameters, which together establish the theoretical basis for process optimization.^20^ In particular, the solubility parameter acts as a pivotal factor in the successful isolation of the solutes within solvent system. Therefore, a comprehensive thermodynamic assessment of the solutes is indispensable for the rational design of DESs.^21^ The *σ*-profile offers a quantitative representation of the local surface polarity distribution of molecules, which is generally partitioned into three distinct zones (HBD, non-polar and HBA regions). As the characteristic marker of EO-LC, LIG functions as both a critical quality-control indicator and the primary material basis for its pharmacological activities.^22^ The majority of the *σ*-profile of LIG, as depicted in Fig. 1A, exhibited predominant localization in the nonpolar region. This molecular feature was strongly correlated with the inherent structural components of LIG, namely benzene rings and vinyl groups. Hence, LIG was a hydrophobic molecule. Simultaneously, the prominent peaks for LIG were observed within the polar domains corresponding to the HBA regions, confirming its inherent HBA characteristics.

Furthermore, the *σ*-potential serves as a crucial thermodynamic descriptor for quantitatively evaluating the intermolecular affinities among different components. In this context, a highly negative *µ*(*σ*) value denotes robust attractive intermolecular forces, whereas a positive value points toward dominant repulsive interactions. Following the segmentation criteria for the *σ*-profile, the *σ*-potential plot can be similarly partitioned into three distinct sections: the HBD, non-polar, and HBA zones.^23^ As illustrated in Fig. 1B, the substantially negative *σ*-potential of LIG within the HBD domain implied an enhanced capability to interact with HBDs. Conversely, positive values with favorable magnitudes were consistently observed in the HBA region for LIG, which implied the presence of repulsive forces between LIG and HBA. Consequently, LIG exhibits higher solubility in DESs with strong HBD characteristics and hydrophobic groups, which is more favorable for the extraction of EO-LC.

### 3.2 Thermodynamic analysis of the candidate deep eutectic solvents

The *σ*-profile and the *σ*-potential for HBAs and HBDs were presented in Fig. 1. As typical HBAs, BET and ChCl showed comparable physicochemical characteristics, while LA, CA, BUT and other compounds acted as representative HBDs. The greater absolute value in the HBD region of *σ*-profile demonstrated that LC and LA exhibit prominent proton donor ability. LC and LA were likely to be the most promising HBDs. However, DESs, an emerging class of green solvents, were typically formulated *via* hydrogen-bonding networks between HBAs and HBDs. The fundamental physicochemical traits of DESs were largely dictated by the inherent characteristics and mutual interaction modes of their constituent HBAs and HBDs.^24^ Consequently, screening the ideal HBA/HBD pairing is crucial for maximizing the yields of EO-LC. The *σ*-profile distributions for DES1 ∼ DES16 at a molar ratio of 2 : 1 were presented in Fig. S1, alongside the corresponding polar region peaks detailed in Table S5. Analysis of these profiles revealed that all investigated DESs display pronounced peaks within the non-polar region, closely mirroring the *σ*-profile of LIG. This similarity indicated that van der Waals forces were a crucial driving force in the molecular interactions, thereby favorably facilitating the extraction of EO-LC.

**Fig. 2:**
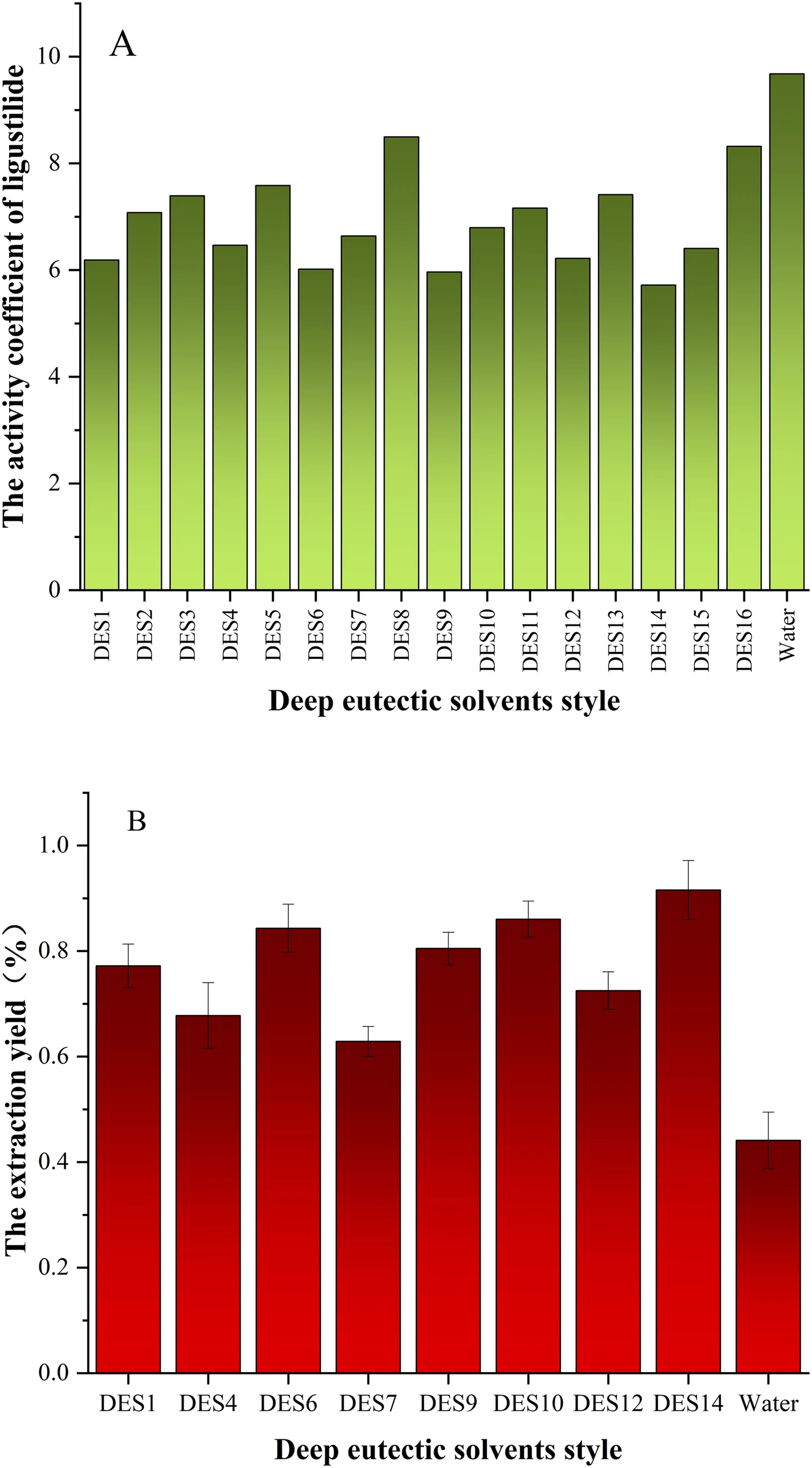
(A) The activity coefficient of ligustilide in candidate deep eutectic solvents. (B) The extraction yield of essential oil from *Ligusticum chuanxiong* using candidate deep eutectic solvents (extraction conditions: molar ratio of 2 : 1, water content of 50 wt%, liquid-to-material ratio of 8 : 1 mL g^−1^, extraction time of 3.0 h).

However, the wide variety of candidate solvent systems greatly reduces screening efficiency, thereby impeding high-throughput precise screening. As a fundamental thermodynamic parameter, the activity coefficient describes the deviation of real solutions from ideal behavior. It can quantitatively characterize intermolecular interaction strength and dissolution affinity. A smaller value suggests superior solubility and extraction capacity of the solute in the candidate solvent system.^25^ In this study, the activity coefficients of LIG in a series of candidate DESs with a molar ratio of 2 : 1 were systematically simulated at 25 °C. As presented in Fig. 2A, the activity coefficients of LIG in the candidate DESs followed a distinct ascending order: DES14 < DES9 < DES6 < DES1 < DES12 < DES4 < DES7 < DES10 < water. Meanwhile, the activity coefficients of LIG in all screened DESs were greater than 1, suggesting the weak intermolecular interactions between DESs and LIG as well as the high volatility of EO-LC. In comparison, the interaction between DES14 and LIG was stronger, which is beneficial to the diffusion and mass transfer of LIG during the extraction process.

### 3.3 Screening for the optimum deep eutectic solvent

Since COSMO-RS is a theoretical simulation and prediction method that involves inherent idealized assumptions and model deviations. Experimental verification is essential to calibrate the simulated results and reveal the actual rules of dissolution and extraction. As presented in Fig. 2B, the extraction performance trends of all candidate DESs were fully consistent with the theoretical predictions derived from COSMO-RS, except DES6 and DES10. Notably, the EO-LC yields obtained using DESs were markedly superior to those of the conventional SD method, which yielded only 0.44%. The underlying mechanism for the enhanced extraction efficiency of DESs may be explained by a dual-action synergistic mechanism. On the one hand, DESs can establish weak intermolecular interactions with the constituents of EO-LC, effectively reducing mass transfer resistance and accelerating the diffusion of target active ingredients from the plant matrix to the extraction solvent.^26^ On the other hand, DESs readily form stable and strong H-bonds with macromolecular components of plant cell walls, including pectin, lignin and various polysaccharides. These robust intermolecular interactions disrupt the compact and rigid architecture of plant cell walls, leading to structural loosening and morphological remodeling.^27^ The combined effects of cell wall structure destruction and accelerated target component diffusion significantly boost the interfacial mass transfer efficiency during the extraction process, thereby substantially improving the extraction efficiency of EO-LC.^28^ Especially, DES6, DES10 and DES14 showed particularly excellent extraction efficiency. This phenomenon was mainly ascribed to the appropriate acidity of CA and LA, which promoted plant cell wall disruption and dissolution.^27,28^ Among all screened DESs, DES14 achieved the highest extraction yield of EO-LC (0.91%), which was 2.07-fold higher than that of the conventional SD method. This superior extraction performance of DES14 can be largely attributed to the strong intermolecular interactions between DES14 and LIG, consistent with the lowest activity coefficient of LIG observed in all screened DESs.

### 3.4 Analysis and discussion of single-factor experimental results

#### 3.4.1 Effects of molar ratio on the extraction yield

**Fig. 3:**
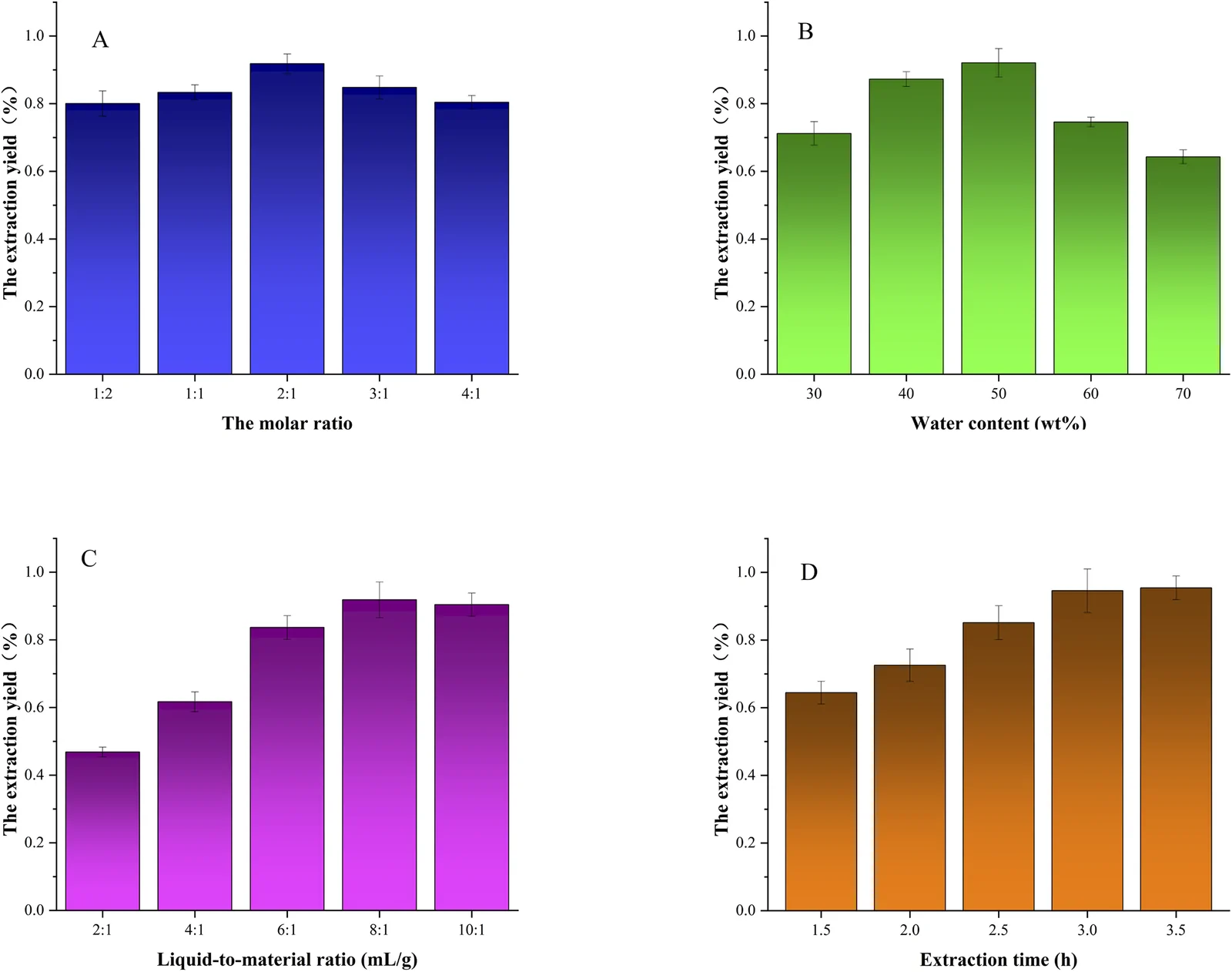
(A) Effects of the molar ratios on the extraction yield (extraction conditions: water content of 50 wt%, liquid-to-material ratio of 8 : 1 mL g^−1^, extraction time of 3.0 h), (B) effects of water contents on the extraction yield (Extraction conditions: molar ratio of 2 : 1, liquid-to-material ratio of 8 : 1 mL g^−1^, extraction time of 3.0 h), (C) effects of liquid-to-material ratio on the extraction yield (extraction conditions: molar ratio of 2 : 1, water content of 50 wt%, extraction time of 3.0 h), (D) effects of extraction time on the extraction yield (extraction conditions: molar ratio of 2 : 1, water content of 50 wt%, liquid-to-material ratio of 8 : 1 mL g^−1^).

The extraction efficacy of DESs is directly governed by the thermodynamic behavior and physicochemical characteristics. The extensive H-bond network inherent to DESs typically results in high viscosity and poor fluidity. These physical and chemical characteristics can severely restrict mass transfer during the practical extraction process.^29^ Consequently, the rational optimization of the molar ratio between HBD and HBA is a prerequisite for tailoring DESs with ideal extraction performance. As illustrated in Fig. 3A, the extraction yield of EO-LC exhibited a distinct bell-shaped trend, initially rising to a peak and subsequently declining as the molar ratio increased. Specifically, when the molar ratio of DES14 was below 2 : 1, increasing the HBD proportion enhanced the H-bond binding capacity and H^+^ concentration of DESs. This facilitation likely intensified the disruption of the cell wall structure of *L. chuanxiong*, accelerating the release and dissolution of EO-LC.^30^ Conversely, the molar ratio exceeding 2 : 1 negatively impacted the extraction kinetics. This reduction was attributed to the increase of system viscosity and steric hindrance, which hindered solvent penetration.

#### 3.4.2 Effects of water content on the extraction yield

Water functions as an essential structural mediator in DESs and strongly influences the physicochemical properties. In particular, the viscosity of DESs exhibits a non-linear dependence on water content.^31^ As shown in Fig. 3B, the extraction yield of EO-LC initially increased and subsequently declined with increasing water proportion, reaching a maximum value of 0.93% when DES14 contained 50 wt% water. When the water contents was below 50 wt%, the incorporation of water effectively attenuated the viscosity of DES14. This enhancement in fluid dynamics promoted more efficient mass transfer between the plant matrix and the extraction solvent.^32^ When the water content exceeded 50 wt%, the physicochemical properties of DES14, such as viscosity and density, tend to stabilize. However, an excessive water simultaneously disrupted the H-bonding network that maintains the structural integrity of DES14. The weakening of these intermolecular interactions diminished the capacity of DES14 to solubilize cell-wall components (*e.g.*, polysaccharides, pectin, and cellulose), thereby limiting the penetration of DESs into the plant matrix and hindering the release of EO-LC.^33^ These results indicated that moderate water content optimizes both DESs fluidity and H-bond interactions.

#### 3.4.3 Effects of liquid-to-material ratio on the extraction yield

The liquid-to-material ratio is a critical parameter governing the thermodynamics and kinetics of the extraction process. Fundamentally, it determines the concentration gradient between the interior of plant powder and DESs, which constitutes the primary driving force for the mass transfer of target compounds.^34^ As illustrated in Fig. 3C, the extraction yield of EO-LC exhibited a robust positive correlation as the liquid-to-material ratio increased from 2 : 1 to 8 : 1 mL g^−1^. When liquid-to-material ratio below 8 : 1 mL g^−1^, the insufficient amount of DESs likely led to rapid saturation and high viscosity, which hindered the complete infiltration of DESs into plant powder. Increasing liquid-to-material ratio not only ensured adequate wetting of plant powder but also maximized the concentration gradient, thereby facilitating the efficient elution of EO-LC into DESs phase. However, as the liquid-to-material ratio was further increased to 8 : 1–10 : 1 mL g^−1^, the slight reduction of the extraction yield of EO-LC was observed. This decline may be attributed to excessive DES14 dissipating the energy supplied for cell wall disruption.^35^

#### 3.4.4 Effects of extraction time on the extraction yield

Extraction time is a crucial operational parameter that significantly influences both the extraction efficiency and the quality of EO-LC. It is related to that extraction time modulates mass transfer driving force and diffusion equilibrium in the extraction processes.^36^ As shown in Fig. 3D, the EO-LC yield increased progressively as the extraction time was extended from 1.5 to 3.0 h. This trend can be attributed to the gradual diffusion of EO-LC from the plant matrix into solvent system, which enhanced the overall extraction efficiency. However, when the extraction time was further prolonged to 3.0 h, the yield showed only marginal variation. This phenomenon can be attributed to the gradual decrease in the concentration gradient between the plant matrix and solvent system as the extraction approached equilibrium. Consequently, the driving force for mass transfer diminished, resulting in a plateau in the EO-LC yield. Therefore, extending the extraction time beyond 3.0 h provided negligible improvements in the EO-LC yield while unnecessarily increasing energy consumption and operational costs.^37^

### 3.5 Response surface results analysis

RSM is a robust statistical method for data modeling through polynomial fitting. It can intuitively reveal the effects of key variables on the extraction yield of EO-LC, clarify the primary and secondary relationships of process factors, and screen out the optimal extraction parameter combination.^38^ The experimental design and factor levels were detailed in Table 1, with the resulting regression model for the yield of EO-LC (*Y*_1_, %) described by eqn (3):

3 *Y*_1_ = 1.08 − 0.2021*A* + 0.0516*B* + 0.0378*C* − 0.0462*AB* + 0.1349*AC* − 0.0487*BC* − 0.1939*A*^2^ − 0.082*B*^2^ − 0.1109*C*^2^

**Table 1:** Response surface design results for extracting essential oil from *Ligusticum chuanxiong*

No.	Variables			Response (%)
	Water content (wt%)	Liquid-to-material ratio (mL g^−1^)	Extraction time (h)	
1	60	6	3.5	0.82
2	50	10	2.5	0.98
3	50	6	2.5	0.8
4	50	8	2.5	0.91
5	60	8	3	0.78
6	50	10	3	1.05
7	40	8	3	1.03
8	50	10	3.5	0.92
9	50	8	3	1.14
10	50	8	2.5	0.86
11	50	8	3	1.07
12	40	6	3	0.79
13	60	8	3.5	0.83
14	50	6	3	0.89
15	40	8	3.5	0.78
16	40	10	3	0.97
17	60	8	3	0.78

*F*-value is utilized to evaluate the relative importance of each variable, with higher *F*-values indicating more pronounced significance. And the statistical relevance is established at a *P*-value threshold of 0.05. As detailed in Table 2, the analytical results of factor impacts on the EO-LC yield revealed that the model is highly reliable (*F* = 7.79, *P* < 0.05). This robust fit was further substantiated by a non-significant lack-of-fit (*F* = 3.22, *P* > 0.05), suggesting no appreciable model error. The relatively high coefficients of determination (*R*^2^ = 0.9385 and *R*_adj_^2^ = 0.9024) demonstrated a significant consistency between the experimentally determined extraction yield of EO-LC and the model predictions. Accordingly, the established regression equation was a dependable tool for interpreting extraction variations. According to *F*-value assessments, the variables were prioritized as *A* > *B* > *C*, while quadratic interactions followed the sequence *AC* > *BC* > *AB*. ANOVA results confirmed that the linear terms (*A*, *B*) and quadratic terms (*AB*, *AC*, *A*^2^, *B*^2^, *C*^2^) exerted significant effects on the EO-LC yield (*P* < 0.05), whereas other parameters remained statistically negligible.

**Table 2:** Variance analysis of regression model

Parameter	Sum of squares	Degree of freedom	Mean square	*F*-value	*P*-value
Model	0.1958	9	0.0218	7.79	0.0065
*A*	0.0425	1	0.0425	15.22	0.0059
*B*	0.0176	1	0.0176	6.28	0.0406
*C*	0.0074	1	0.0074	2.65	0.1475
*AB*	0.0046	1	0.0046	1.64	0.0411
*AC*	0.0249	1	0.0249	8.91	0.0204
*BC*	0.0075	1	0.0075	2.68	0.1453
*A* ^2^	0.0875	1	0.0875	31.32	0.0008
*B* ^2^	0.0220	1	0.0220	7.85	0.0264
*C* ^2^	0.0472	1	0.0472	16.90	0.0045
Residual	0.0196	7	0.0028		
Lack of fit	0.0159	4	0.0040	3.22	0.1823
Pure error	0.0037	3	0.0012		
Cor total	0.2154	16			

**Fig. 4:**
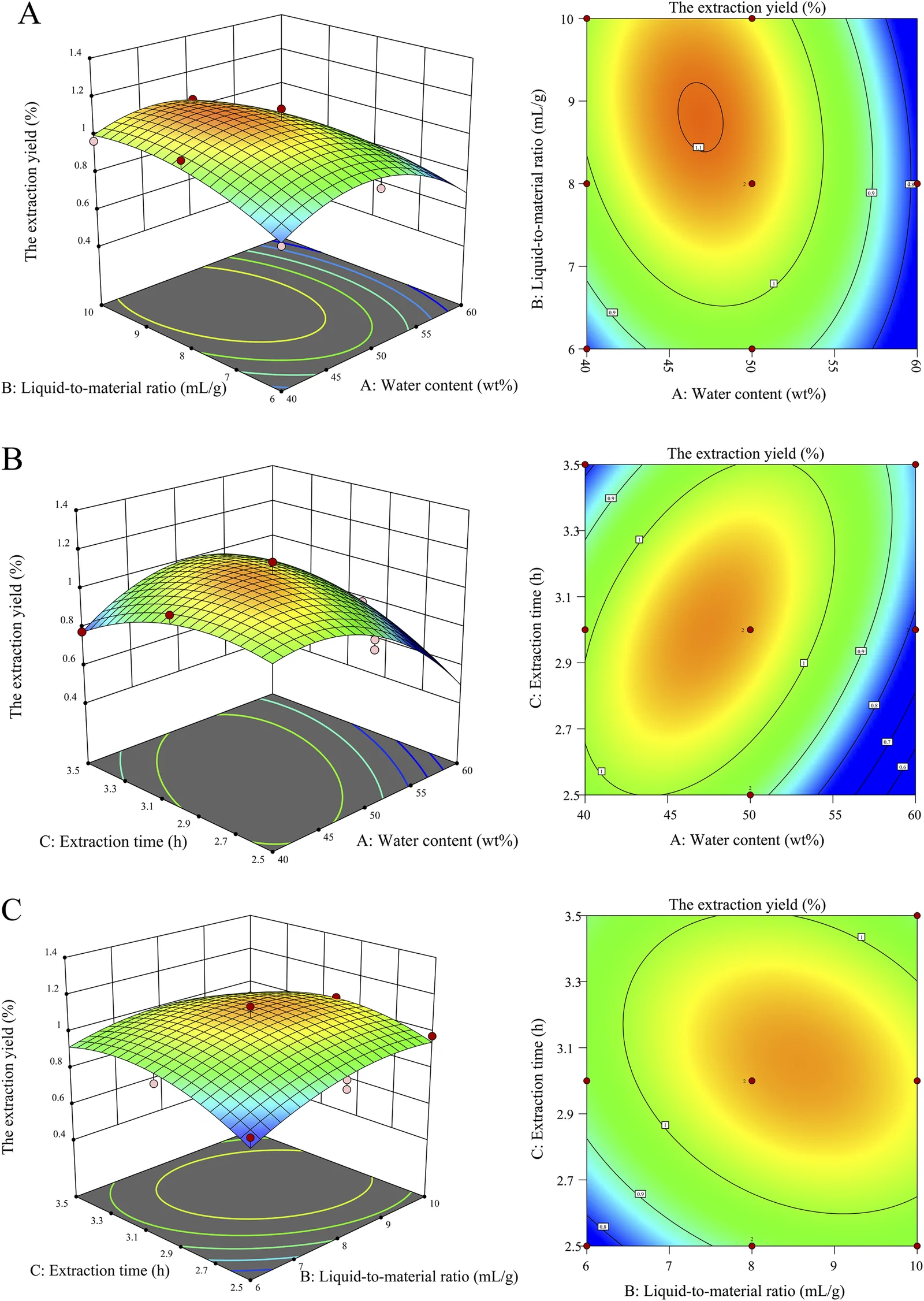
Response surface graph and contour plot reflect the interactive effects of extraction parameters on the extraction yield. (A) Water content and liquid-to-material ratio, (B) water content and extraction time, (C) liquid-to-material ratio and extraction time.

The steepness of response surface curves reflect the influence magnitude of two factors on the extraction yield of EO-LC. The pronounced curvature of the response surfaces, coupled with the dense contour lines, effectively highlighted the significant interdependencies between the investigated variables.^37^ As shown in Fig. 4A, the response surface showed a clear slope within the ranges of 45 ∼ 60 wt% water content (A) and 6 ∼ 9 mL g^−1^ liquid-to-material ratio (B), with dense elliptical contour lines. This demonstrated that the marked interaction between A and B, significantly affected the extraction yield of EO-LC. In contrast, Fig. 4B exhibited an obvious slope within the ranges of 45 ∼ 60 wt% water content and 2.5 ∼ 3.1 extraction time. The elliptical and dense contour lines reflected a significant interaction between A and C, suggesting that their interaction markedly influenced the extraction yield of EO-LC. Similarly, in Fig. 4C, the relatively flat response surface, accompanied by sparse and nearly circular contour lines, indicated the absence of a significant interactive effect between A and B on the extraction yield of EO-LC.

According to the RSM optimization, the maximum predicted theoretical yield was 1.09% under the following optimal experimental conditions: a water content of 48.3 wt%, a liquid-to-material ratio of 8.5 : 1 mL g^−1^, and an extraction time of 2.9 h. Considering practical operation conditions, the optimized extraction process for EO-LC was set as follows: water content of 48 wt% (A), liquid-to-material ratio of 8.5 : 1 mL g^−1^ (B), and extraction time of 2.9 h (C). Under these optimal conditions, the experimental extraction yield of EO-LC reached 1.14 ± 0.07 mg g^−1^, which was in good agreement with the predicted value of 1.09%. With the relative error of 4.58%, this experimental result verified the accurate prediction of the established RSM model. Compared with the conventional SD method (0.44%), the EO-LC yield of DES14 was increased by 1.59-fold. Therefore, DES14 exhibited the superior efficacy in enhancing the extraction yield of EO-LC.

### 3.6 Composition analysis of essential oil from *Ligusticum chuanxiong*

The chemical components of EO-LC were identified by comparison with the NIST 17.0 database, and the relative contents were determined using the peak area normalization method. Total ion chromatogram of EO-LC was shown in Fig. S2. As presented in Table 3, a total of 37 constituents were identified in EO-LC extracted by DES14. The main chemical categories of EO-LC were composed of phthalide lactone compounds, terpenes and alcohols, which collectively contributed to the diverse biological activities of EO-LC. Compared with EO-LC of SD group, EO-LC of DES14 exhibited significant diversity in the chemical composition of EO-LC, with 12 additional volatile components including β-terpineol, β-elemene, α-cedrene, eudesmol and α-selinene. The major chemical components of ligustilide, senkyunolide A, butylidenephthalide and butylphthalide were the material basis for the antioxidant and neuroprotective activities of EO-LC.^4^ Screening the key components with a threshold >2%, the major chemical components of EO-LC in DES14 group were (Z)-ligustilide (46.39%), senkyunolide A (13.82%), butylidenephthalide (6.76%), butylphthalide (4.68%), β-eudesmene (3.71%), α-terpineol (2.65%), and terpinen-4-ol (2.03%). Notably, the relative content of phthalide lactone compounds in EO-LC of DES14 group reached 72.7%, which was significantly higher than that of SD group (60.01%). Among them, (Z)-ligustilide, senkyunolide A, butylidenephthalide, and butylphthalide were the key contributors to the observed difference. The excellent biological activity of EO-LC is primarily ascribed to its high abundance of phthalide lactone constituents, including (Z)-ligustilide, senkyunolide A and butylphthalide. Extensive studies have demonstrated that these bioactive compounds exert neuroprotective effects through the synergistic modulation of multiple signaling pathways and physiological processes.^39–41^ As observed in the high-temperature elution region of the GC-MS chromatogram, SD group contained a substantial number of unidentified components. These results demonstrated that DESs can not only facilitate the efficient extraction of EO-LC but also eliminate interfering impurities, thereby enhancing the overall quality of the target extract. Therefore, EO-LC of DES14 group was a promising candidate for the development of aromatic therapeutics and functional dietary supplements. The establishment of DESs-assisted extraction technology for EO-LC was economically valuable for the extensive utilization of *L. chuanxiong*.

**Table 3:** Chemical components of essential oil from *Ligusticum chuanxiong*

NO.	Retention time (min)	Molecular weight	Molecular formula	Compound	Relative content (%)			
					DES14 group	Similarity	SD group	Similarity
1	4.302	136.23	C_10_H_16_	α-Phellandrene	0.04	94	—	
2	4.340	136.23	C_10_H_16_	4-Carene	0.12	96	—	
3	4.436	134.22	C_10_H_14_	*p*-Cymene	0.2	97	0.04	95
4	4.498	136.23	C_10_H_16_	d-Limonene	0.35	98	0.78	96
5	4.849	136.24	C_10_H_16_	γ-Terpinene	0.8	96	0.11	97
6	5.218	136.23	C_10_H_16_	Isoterpinolene	0.73	98	0.19	97
7	5.904	154.25	C_10_H_18_O	1-Terpinenol	0.6	95	—	
8	6.103	154.25	C_10_H_18_O	β-Terpineol	0.22	96	—	
9	6.189	150.26	C_11_H_18_	5-Pentylcyclohexa-1,3-diene	0.8	97	0.04	97
10	6.560	154.25	C_10_H_18_O	Terpinen-4-ol	2.03	96	0.56	96
11	6.754	154.25	C_10_H_18_O	α-Terpineol	2.65	97	0.14	96
12	6.807	154.25	C_10_H_18_O	γ-Terpineol	0.19	96	0.22	94
13	8.334	150.17	C_9_H_10_O_2_	*p*-Vinylguaiacol	0.02	97	0.74	98
14	8.854	150.13	C_8_H_6_O_3_	1,4-Cyclohexadiene-1,2-dicarboxylic anhydride	0.46	96	0.30	93
15	8.925	162.23	C_11_H_14_O	Valerophenone	0.33	95	0.19	95
16	9.205	186.29	C_11_H_22_O_2_	Ethyl 4-decenyl carbonate	0.16	96	—	
17	9.381	204.35	C_15_H_24_	β-Elemene	0.21	95	—	
18	9.897	204.35	C_15_H_24_	Cubenene	1.07	96	0.15	96
19	10.088	204.35	C_15_H_24_	β-Caryophyllene	0.38	97	—	
20	10.280	220.35	C_15_H_24_O	β-Copaen-4α-ol	0.23	91	—	
21	10.496	204.35	C_15_H_24_	α-Cedrene	0.39	97	—	
22	10.743	204.35	C_15_H_24_	β-Eudesmene	3.71	93	0.49	96
23	10.817	204.35	C_15_H_24_	α-Selinene	0.88	95	—	
24	11.829	220.35	C_15_H_24_O	Espatulenol	0.26	92	1.22	91
25	11.936	222.37	C_15_H_26_O	Eudesmol	0.57	96	—	
26	12.107	136.23	C_10_H_16_	Geranyl-α-terpinene	0.31	95	—	
27	12.477	222.37	C_15_H_26_O	Cadinol	0.79	93	0.28	94
28	12.678	190.24	C_12_H_14_O	Butylphthalide	4.68	95	3.42	97
29	12.932	188.22	C_12_H_12_O_2_	Butylidenephthalide	6.76	97	4.25	96
30	13.140	150.26	C_11_H_18_	5-Pentylcyclohexa-1,3-diene	1.6	95	1.41	92
31	13.519	192.25	C_12_H_16_O_2_	Senkyunolide A	13.82	93	12.38	96
32	13.685	190.24	C_12_H_14_O_2_	Z-ligustilide	46.39	94	39.29	95
33	14.30	190.24	C_12_H_14_O_2_	E-ligustilide	1.05	98	0.67	94
34	15.007	242.44	C_16_H_34_O	1-Hexadecanol	0.36	97	0.84	96
35	15.645	254.41	C_16_H_30_O_2_	Palmitoleic acid	0.29	96	0.67	93
36	15.834	256.42	C_16_H_32_O_2_	*n*-Hexadecanoic acid	0.44	91	0.42	91
37	17.528	282.46	C_18_H_34_O_2_	Oleic acid	0.17	94	1.01	89
38	—	—	—	Unknown	5.94		30.19	

### 3.7 Analysis of AChE inhibitory activity and molecular docking

#### 3.7.1 Analysis of AChE inhibitory activity

As a pivotal enzyme governing acetylcholine homeostasis in the central nervous system (CNS), AChE plays a critical role in maintaining cholinergic system function. However, abnormal AChE activity disrupts the delicate homeostatic balance of CNS, thereby contributing to the pathogenesis and progression of mood disorders such as anxiety and depression. Accordingly, restoring central AChE homeostasis *via* the moderate inhibition of AChE by EO has emerged as an increasingly attractive therapeutic strategy for mitigating this disorders.^41^ As exhibited in Fig. S3, DES14 group displayed a significant concentration-dependent ability to inhibit the AChE activity, with IC_50_ value quantified at 41.25 µg mL^−1^. And SD group also have the moderate inhibitory activity for AChE, as evidenced by IC_50_ values of 51.94 µg mL^−1^. In comparison, DES14 group exhibited superior inhibitory activity for AChE.

Phthalide lactone compounds possess diverse pharmacological properties. Besides potent antioxidant and anti-inflammatory activities, they can modulate neuronal physiological functions and exert prominent neuroprotective effects.^4–6^ Ligustilide presented therapeutic effects on SAMP8 mice by improving mitochondrial function and reversing memory loss.^39^ Senkyunolide A alleviated glutamate-induced cytotoxicity in Neuro2a cells through modulation of the JNK/caspase-3 pathway and inhibition of cellular apoptosis.^40^ Butylphthalide conferred neuroprotection by inhibiting neuronal apoptosis, a process mediated through activation of the BDNF/TrkB signaling pathway.^41^ Therefore, EO-LC extracted by DES14 was a promising AChE inhibitor for the development of aromatic therapeutics and functional dietary supplements.

#### 3.7.2 Molecular docking

Molecular docking is an important structure-based drug discovery tool that evaluates the binding stability of candidate active ingredients with target proteins by calculating binding free energies and identifying key intermolecular interactions. It accelerated the screening of potential bioactive components from natural products and facilitated the in-depth elucidation of their pharmacological mechanisms.^42^ As presented in Table 4, the binding energies between the candidate active ingredients and AChE were predominantly lower than −5.0 kcal mol^−1^. Accordingly, the major constituents of EO-LC showed significant inhibitory activities against AChE. Notably, β-eudesmene exhibited the lowest binding energy of −7.1 kcal mol^−1^, confirming its robust binding affinity with AChE. Although LIG exhibited a binding energy of −6.3 kcal mol^−1^, its relatively high abundance in EO-LC compensated for the minor shortcoming. This results implied that LIG still exerted a notable regulatory role in the AChE inhibitory activity. The relative content of phthalide lactone compounds in EO-LC obtained by DES14 was significantly higher than that by the conventional SD method, which provided a reasonable theoretical explanation for its stronger inhibitory activity for AChE.

**Table 4:** Binding energies and active site interactions between key components and AChE

Compound	Relative content (%)	Binding energies (kcal mol^−1^)	Active site interactions
Butylphthalide	4.68	−6.7	LEU380, HIS381, TRP385, ALA530
Butylidenephthalide	6.76	−6.3	HIS405, PRO410, PRO537, LEU540
Senkyunolide A	13.82	−6.9	ALA412, CYS529, ALA530, ASN533, ARG534
Z-ligustilide	46.39	−6.3	LEU380, HIS381, TRP385, GLN527, ALA530
β-Eudesmene	3.71	−7.0	PRO235, PRO410, HIS504, LEU536, PRO537, LEU540
Terpinen-4-ol	2.03	−5.9	VAL12, LYS53, TRP56, LEU178, TRP182

H-bond is a pivotal non-covalent interaction that stabilizes small molecule–protein binding. It can substantially enhance the binding affinity of EO-LC-protein and the structural integrity of the resulting complexes. In this study, the optimal binding conformations of major active ingredient with AChE, obtained from molecular docking, were further analyzed to elucidate the underlying intermolecular interaction patterns. As illustrated in Fig. S4–S9, all candidate active ingredients form extensive hydrogen bonds with amino acid residues of AChE. These interactions were critical forces in maintaining the stable binding of the complexes. For example, Z-ligustilide formed conventional and C⋯H bonds with AChE through amino acid residues such as HIS381, TRP385, ALA530. Butylphthalide established conventional H-bonds and C⋯H bonds with AChE through amino acid residues, namely TRP385 and ALA530. There were also a significant van der Waals force between AChE and the candidate active ingredients, such as LEU380, GLN527, GLN413, ASN233. These results collectively indicated that EO-LC formed hydrogen bonds and hydrophobic interactions with amino acid residues of AChE, including GLN527, HIS381, LEU380, GLN527, *etc.* Consequently, EO-LC was a reversible inhibitor that bound to AChE by non-covalent interactions, forming a reversible enzyme–inhibitor complex.

### 3.8 Mechanistic analysis of deep eutectic solvent extraction

**Fig. 5:**
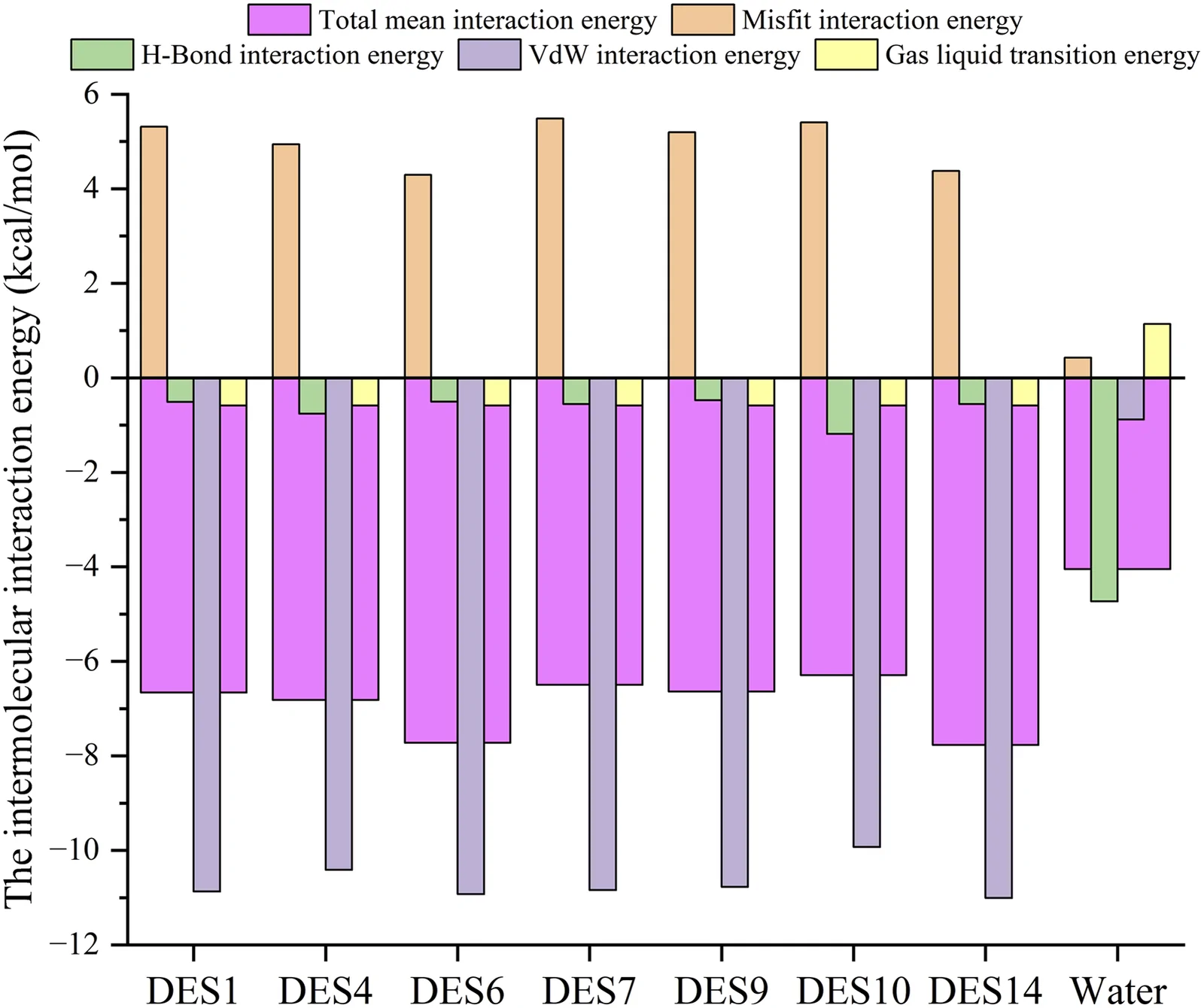
The intermolecular interaction energy between ligustilide and the candidate deep eutectic solvents.

According to the similarity-intermiscibility theory, van der Waals forces are the primary driver of EO extraction with DESs. However, there is currently no evidence available for the extraction mechanism and its primary driving forces. As a computational simulation technique, the COSMO-RS model has emerged as an indispensable tool for elucidating the fundamental behaviors of complex extraction processes.^9^ The total binding energy was more negative when target compound bound the extractant with stronger affinity. And the smaller the negative binding energy, the greater the extraction efficiency and enrichment.^10^ As summarized in Fig. 5, the total interaction energy between LIG and DES14 was lower than that of other DESs, with the corresponding value reaching −7.77 kcal mol^−1^. In addition, the total interaction energy between LIG and water was the weakest, which indicates the poor leaching capacity of water for EO-LC. Therefore, DESs possess a stronger extraction ability for EO-LC than water.

The COSMO-RS model decomposes the total interaction energy into three intermolecular interaction components, namely electrostatic misfit energy, hydrogen bond energy, and van der Waals energy, which collectively determine the dissolution affinity, extraction efficiency and enrichment performance of target compounds in DESs. As in Fig. 5, van der Waals force between LIG and DES14 showed the lowest interaction energy of −11.01 kcal mol^−1^. By contrast, the contribution derived from electrostatic misfit energy, hydrogen bond energy was demonstrated to be comparatively weaker. Therefore, van der Waals forces were the pivotal driving forces that modulated the interactions between EO-LC and DES14. In addition, van der Waals forces were weak intermolecular interactions that are highly sensitive to temperature variations.^43^ High temperature attenuated van der Waals forces, thereby facilitating the desorption of EO-LC, and laying the foundation for subsequent distillation and enrichment operation. Hence, DES14 possessed superior extraction capabilities for EO-LC, with the highest yield of 1.02%. Numerous studies have demonstrated that H-bond acts as the dominant driving force for EO extraction.^11–13^ By contrast, COSMO-RS simulation provided a distinct insight into the extraction mechanism. This finding offered practical guidance for the rational design of DESs to maximize extraction efficiency of EO.

## 4 Conclusion

In this study, the COSMO-RS model and combined with experimental validation was adopted to screen the optimal DES for EO-LC extraction. According to the thermodynamic analysis results from COSMO-RS model, betaine/lactic acid (2 : 1) was identified as the optimum DES among all screened DESs. And the extraction results of the candidate DESs were basically consistent with the theoretical predictions derived from COSMO-RS model.

Additionally, the optimal extraction conditions for EO-LC were established by single-factor experiments and RSM, and as follows: a water content of 48 wt%, a liquid-to-material ratio of 8.5 : 1 mL g^−1^, and an extraction time of 2.9 h, achieving the EO-LC yield of 1.14 ± 0.07%. Meanwhile, the chemical composition of EO-LC was analyzed by GC-MS. Compared with the conventional SD method, DES14 significantly increased the extraction yield of EO-LC, enhanced its chemical diversity, and elevated the relative contents of its major active components.

Furthermore, the aromatic therapeutic potential of EO-LC was systematically performed by AChE inhibitory activity and molecular docking. EO-LC of DES14 exhibited superior AChE inhibitory activity, predominantly mediated by hydrogen-bonding and hydrophobic interactions with key amino acid residues within the enzyme active site. The mechanistic analysis of DES-assisted extraction revealed that van der Waals forces are the primary driving force for the efficient extraction of EO-LC.

The above significant improvements provided robust scientific evidence for the technical superiority of DESs in the EO-LC extraction and the development of aromatic therapeutics. Overall, this work establishes a solid theoretical foundation for the rational design of DESs, while offering valuable insights into the efficient extraction and diversified application of EO-LC. However, the recyclability and reusability of the candidate DESs were not investigated in this study. Additionally, the aromatic therapeutic effects of EO-LC remain to be further validated through *in vivo* animal experiments and clinical trials.

## Author contributions

Zhouwei Hong and Liu Tang: investigation, data curation, writing—original draft preparation; Tong Xia: methodology, software, validation, formal analysis; Yuanguo Xiong: resources; Gang Liu: project administration, funding acquisition, supervision; Lian Liu: writing—original draft preparation; Xu Cai: conceptualization, writing—review and editing.

## Conflicts of interest

None.

## Supplementary Material

RA-016-D6RA04452B-s001

## Data Availability

All data generated and analyzed during this study are available within this article and its supplementary information (SI) files. Supplementary information is available. See DOI: https://doi.org/10.1039/d6ra04452b.
