# Supplementary material for: COSMO-RS guided screening of deep eutectic solvent extraction for essential oil from Ligusticum chuanxiong: optimization, composition analysis, and acetylcholinesterase inhibitory activity
Source: RSC Adv. 2026 Sep 1;16(42):51325–37. doi: 10.1039/d6ra04452b (PMC13531298; doi:10.1039/d6ra04452b)
Supplement: Supplementary file 1 [file RA-016-D6RA04452B-s001.pdf]

Supplementary Information (SI) for RSC Advances.
This journal is © The Royal Society of Chemistry 2026

Electronic Supplementary Material

COSMO-RS guided screening of deep eutectic solvent extraction for essential oil from *Ligusticum chuanxiong*: Optimization, composition analysis, and acetylcholinesterase inhibitory activity

Zhouwei Hong,†[a,c] Liu Tang,†[a] Tong Xia,[a,c] Gang Liu,[a] Yuanguo Xiong,[a] Lian Liu,[b*] Xu Cai,[a,c*]

[a] Pharmaceutical Department, Renmin Hospital of Wuhan University, Wuhan Hubei 430060, China. Email: 2021caixu@whu.edu.cn.

[b] Department of Anesthesiology, Renmin Hospital of Wuhan Universityy, Wuhan Hubei 430060, China. Email: elysiall@whu.edu.cn.

[c] College of Pharmacy, Hubei University of Chinese Medicine, Wuhan Hubei 430065, China. Email: 13638697959@163.com.

† These authors contributed equally to this work.

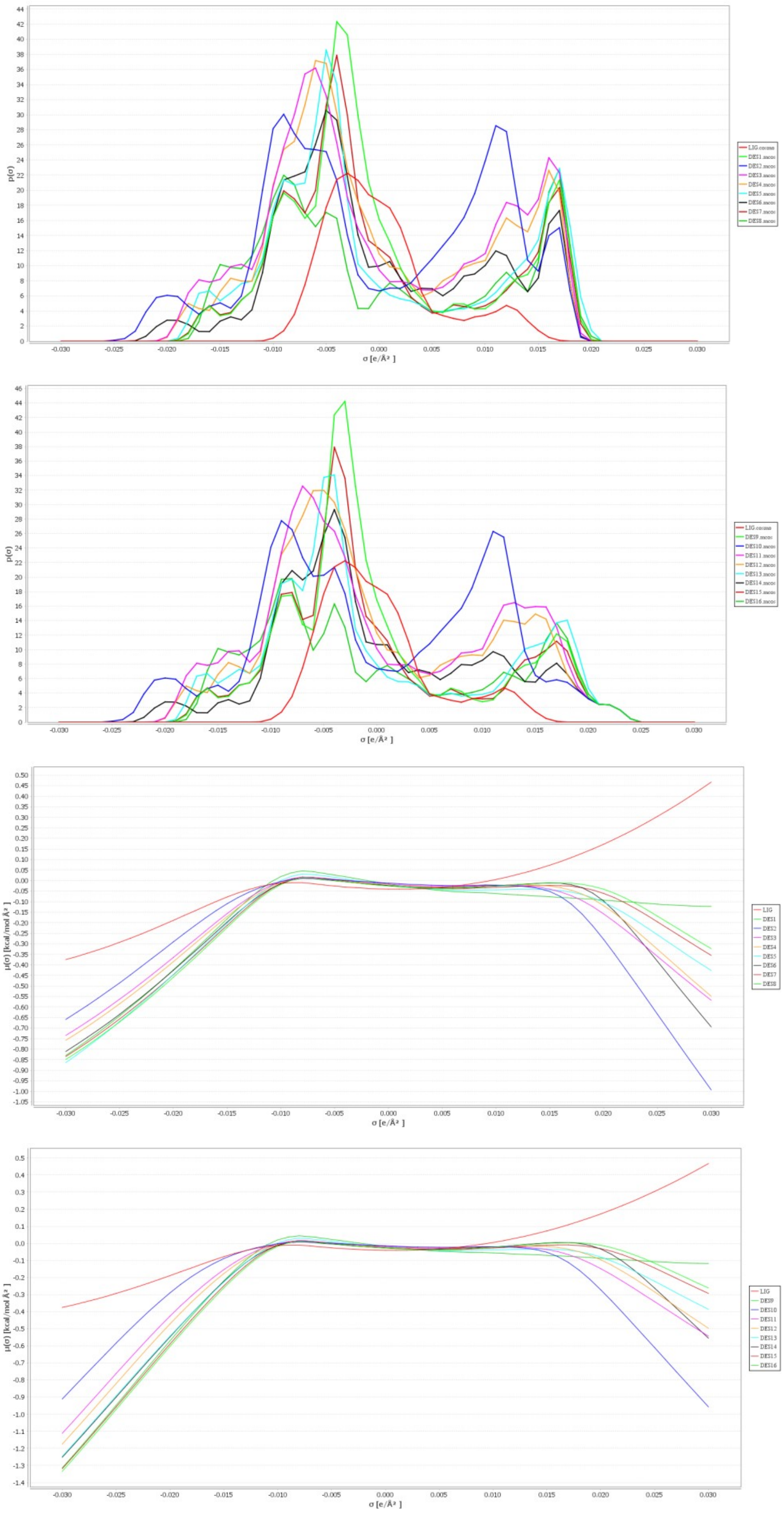

Figure S1. The σ-profile and σ-potential diagram of DES1-DES16

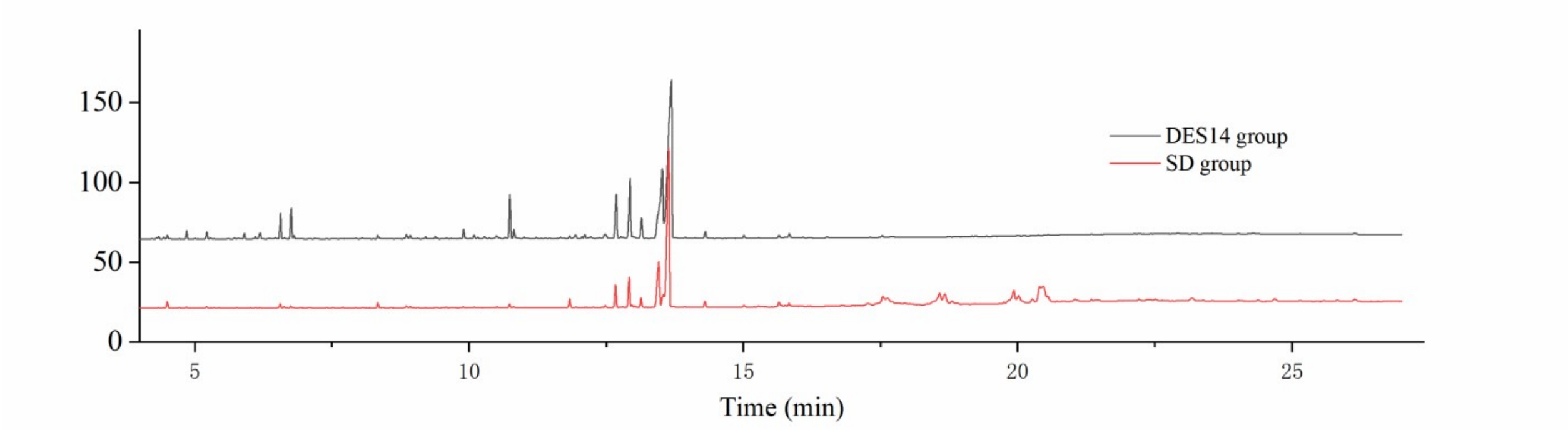

Figure S2. Total ion chromatogram of essential oil from *Ligusticum chuanxiong*

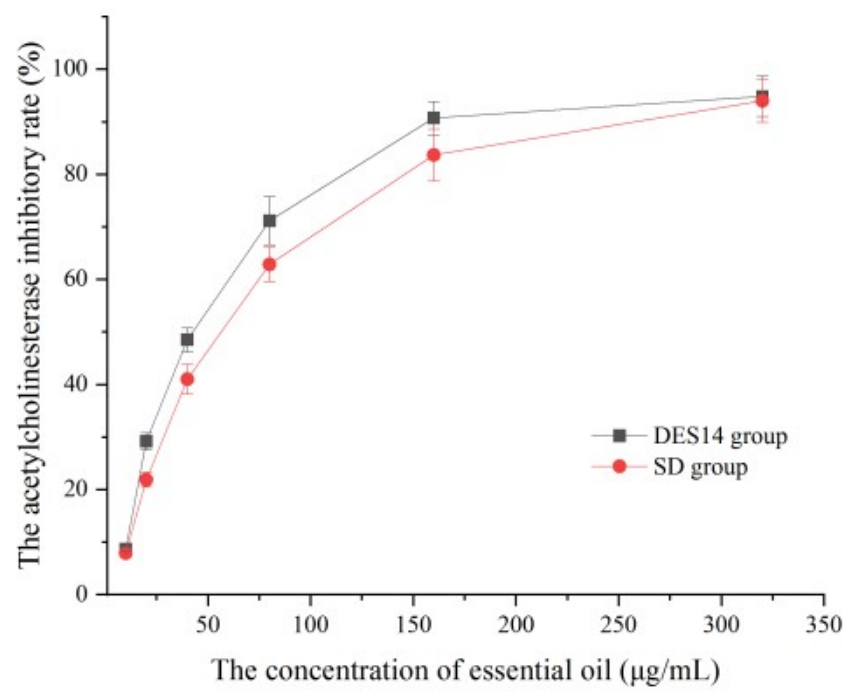

Figure S3. The acetylcholinesterase inhibitory activity of SD group and DES14 group

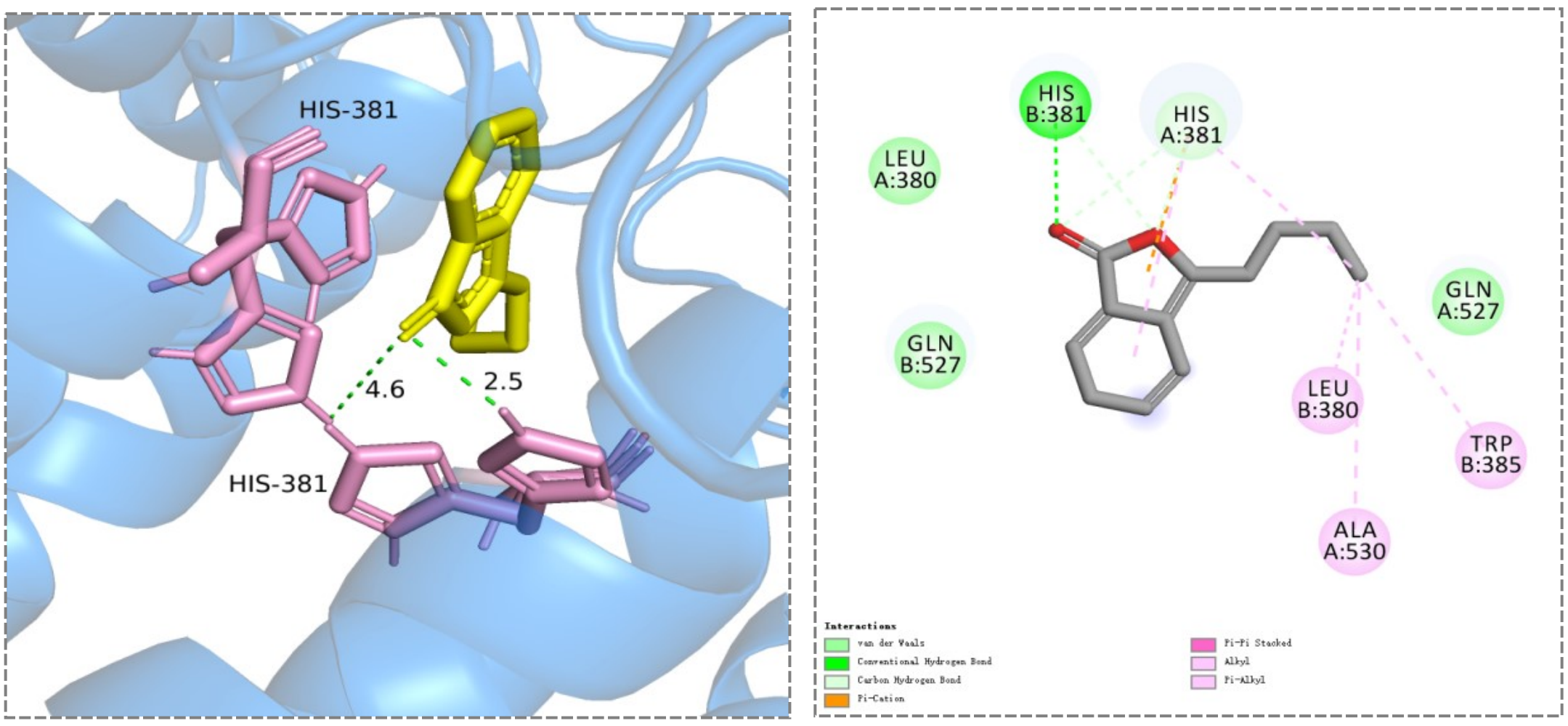

Figure S4. The docking diagram of butyleidenephthalide and 7E3H protein molecules

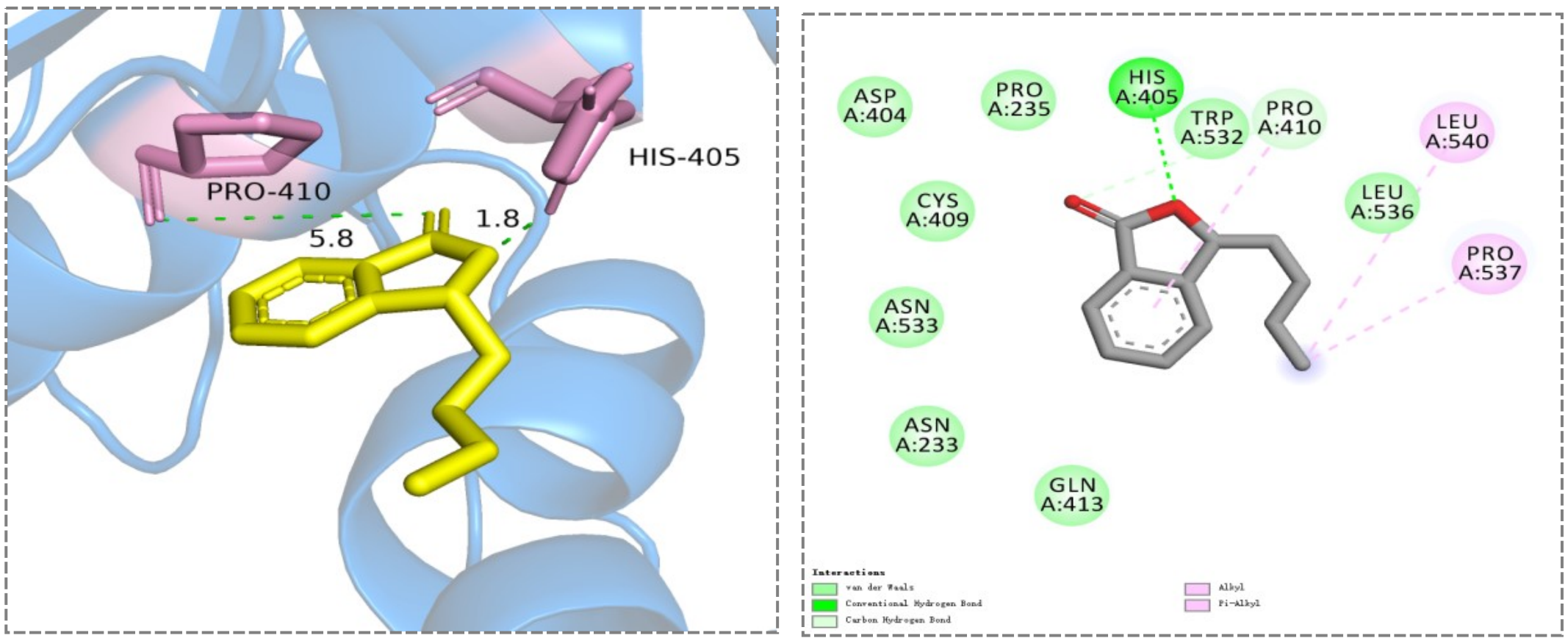

Figure S5. The docking diagram of butylphthalide and 7E3H protein molecules

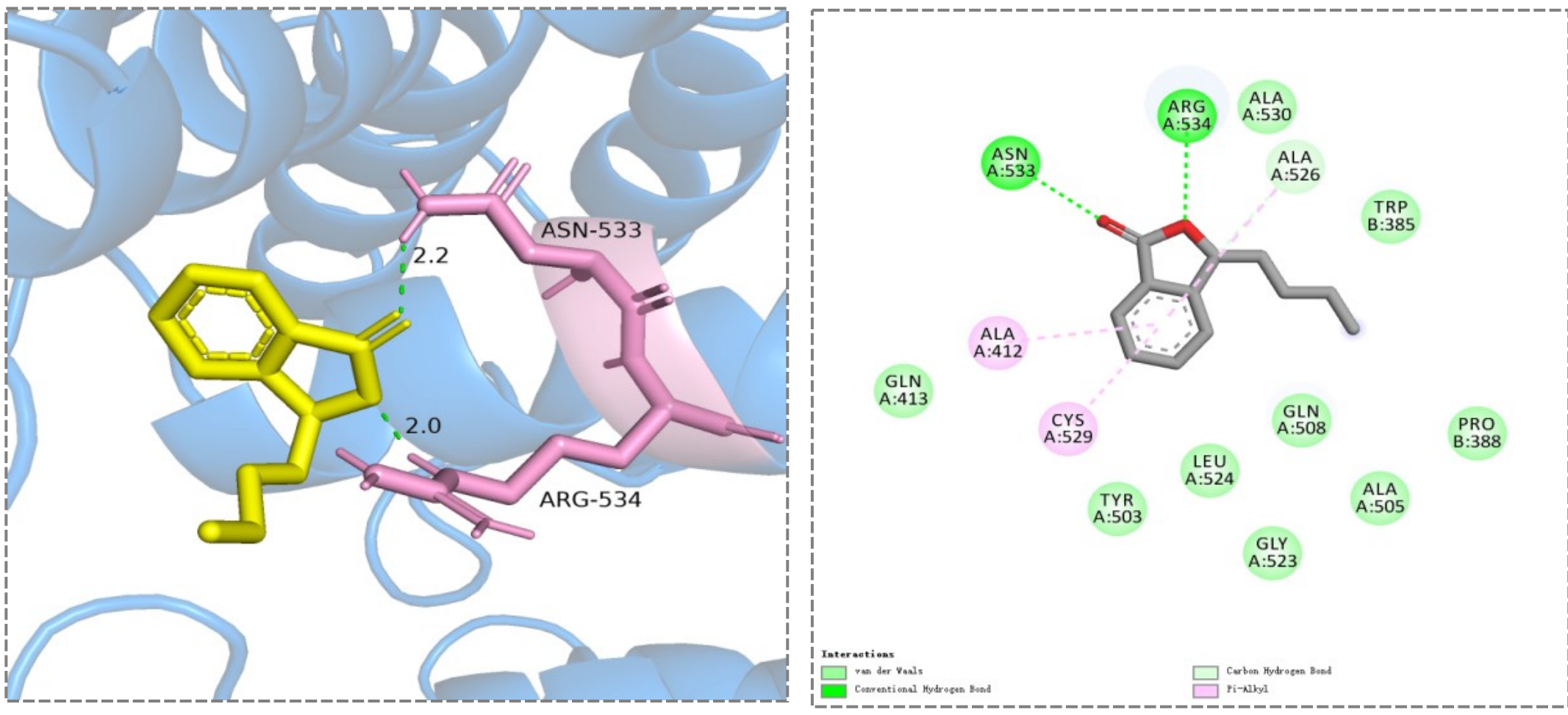

Figure S6. The docking diagram of senkyunolide A and 7E3H protein molecules

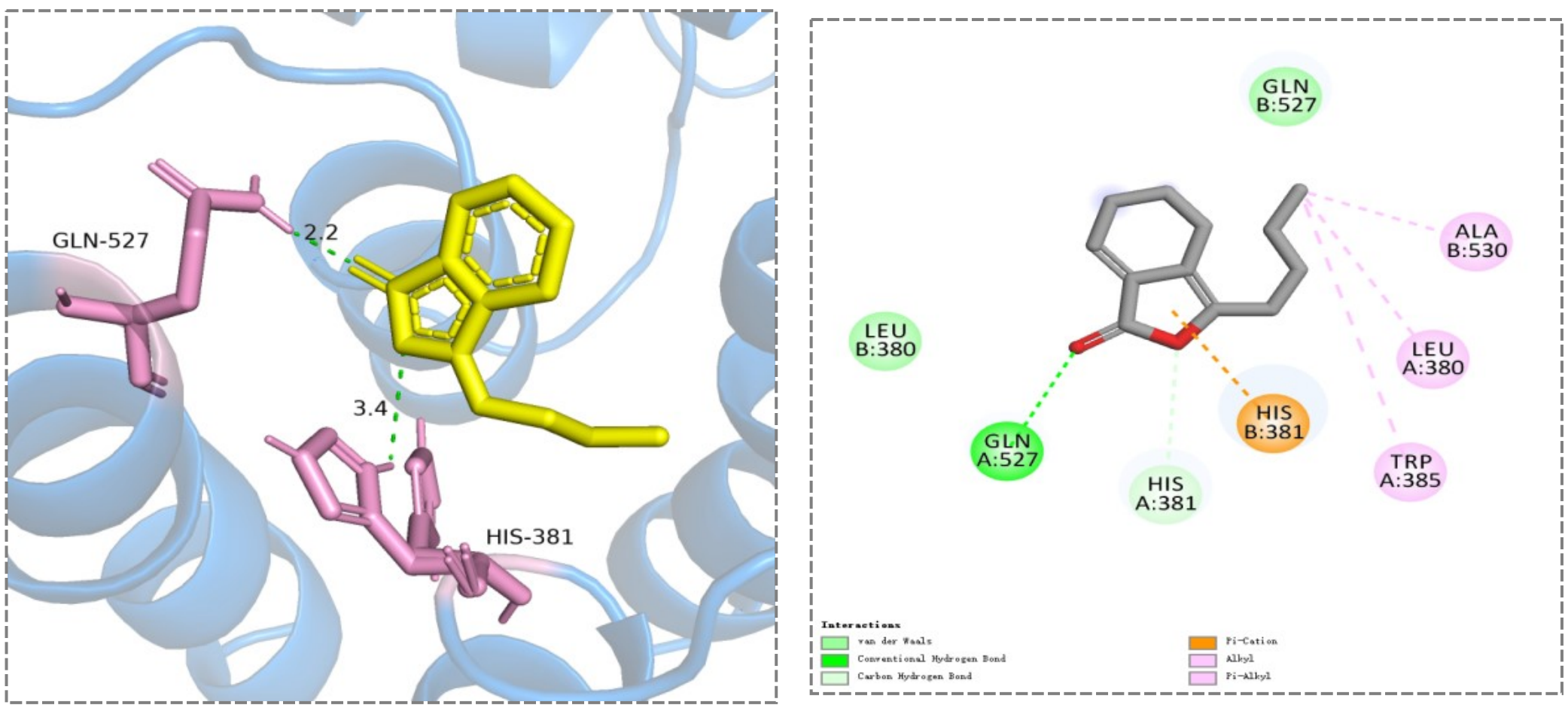

Figure S7. The docking diagram of Z-ligustilide and 7E3H protein molecules

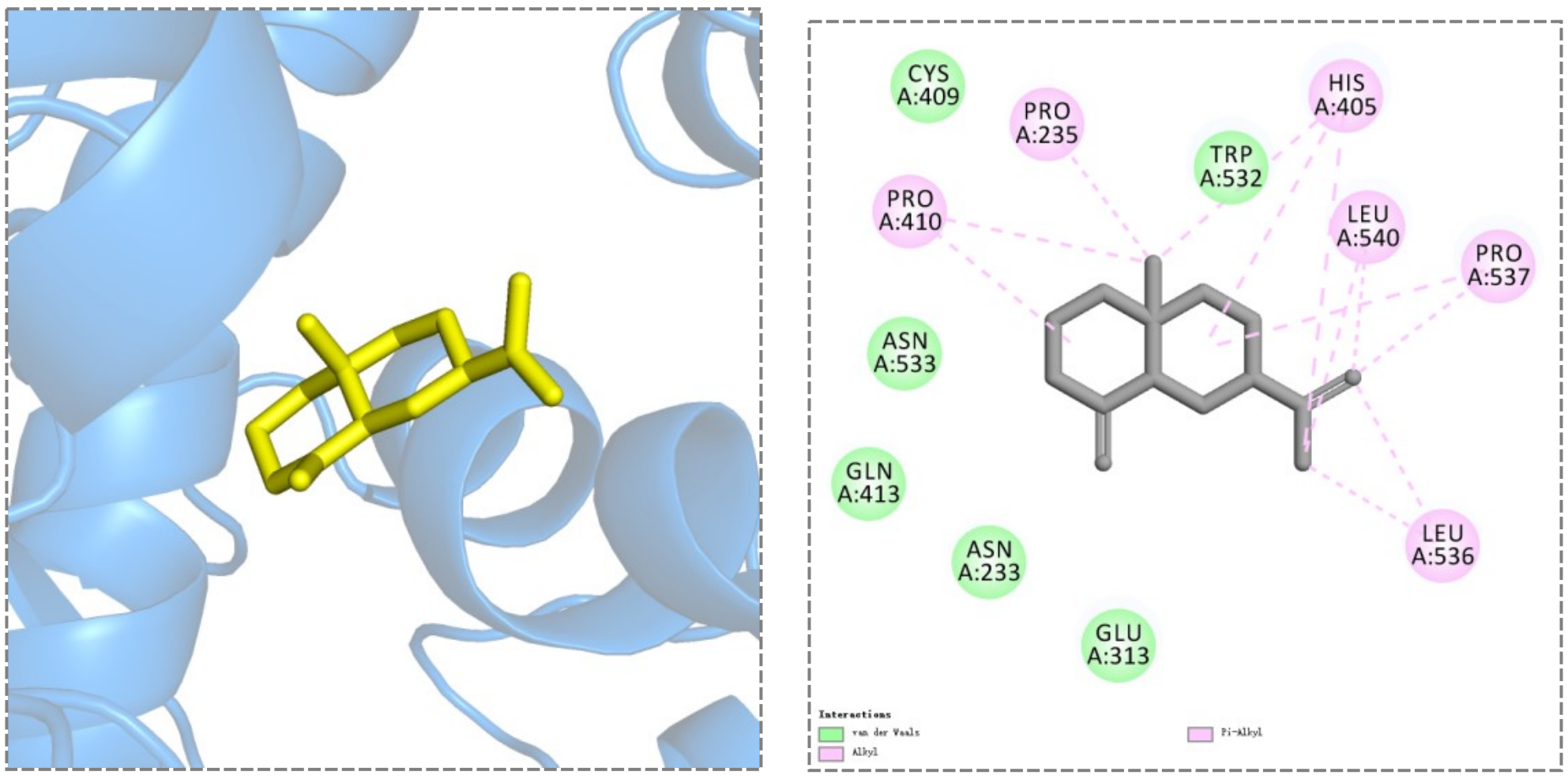

Figure S8. The docking diagram of β-eudesmene and 7E3H protein molecules

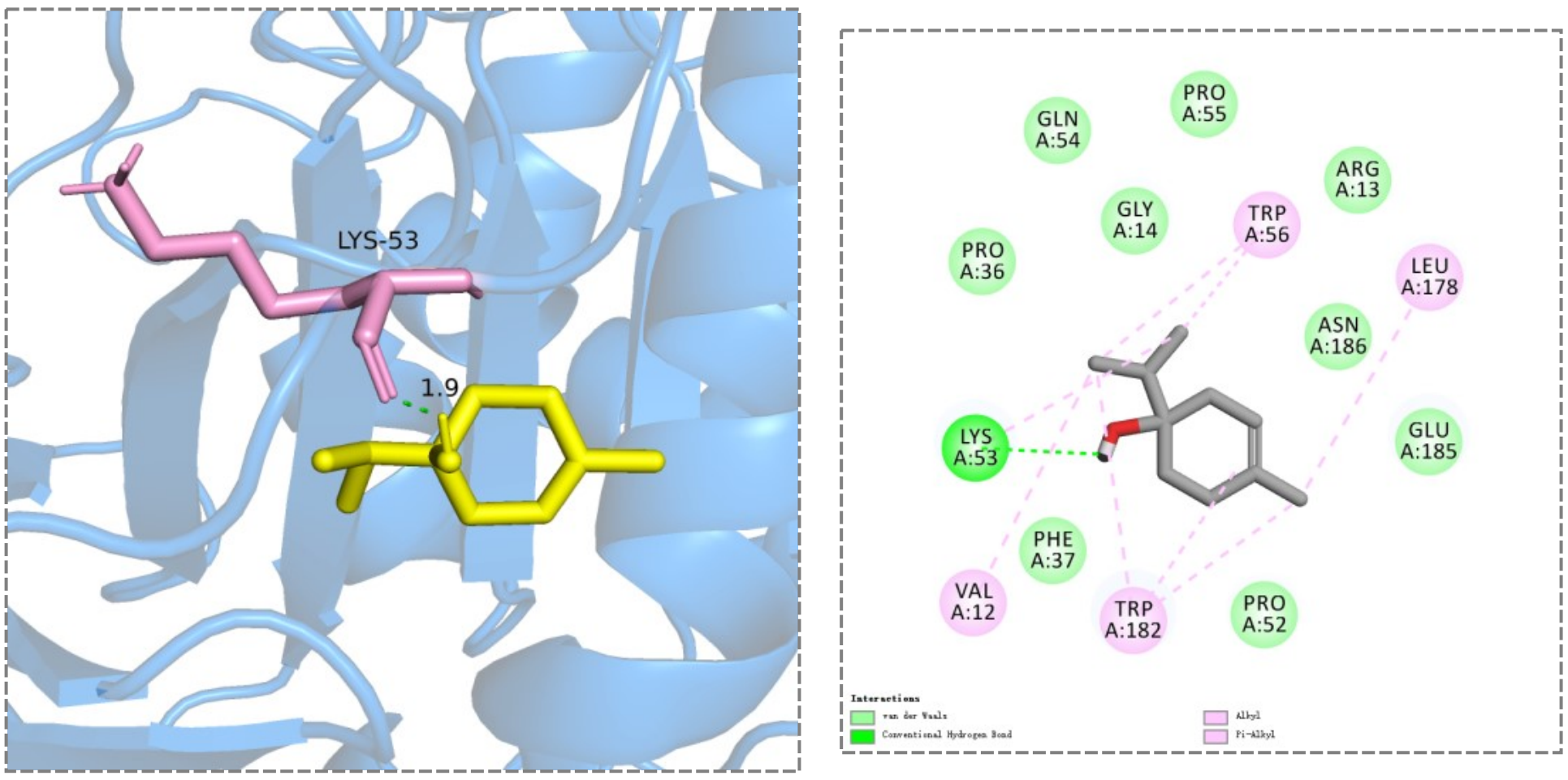

Figure S9. The docking diagram of Terpinen-4-ol and 7E3H protein molecules

Table S1 The structure of dihydromyricetin, HBA and HBD

Type	Name		Structural formula
**Target compound**	Ligustilide	LIG	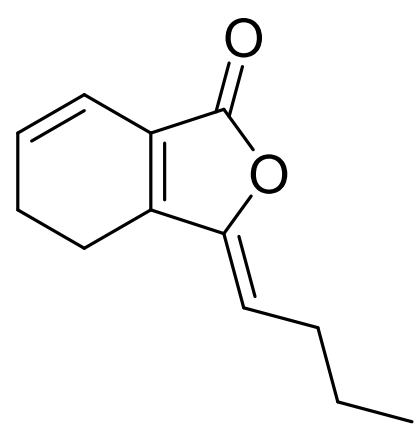
**HBA**	Choline chloride	ChCl	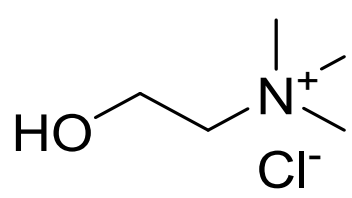
	Menthol	MEN	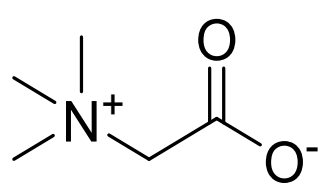

	1,4-butanediol	BUT	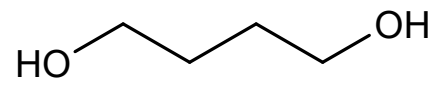
	Citric acid	CA	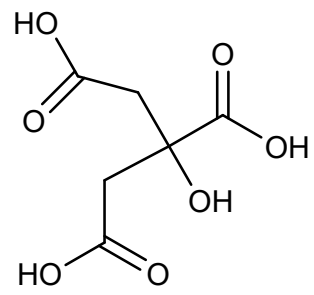
	Glucose	GLU	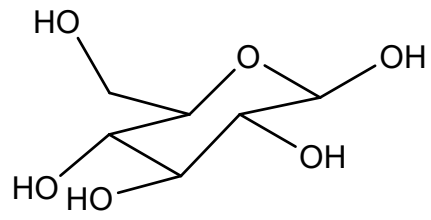
**HBD**	Fructose	Fru	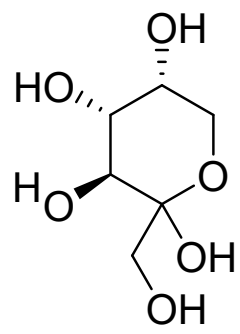
	Glycerol	GLY	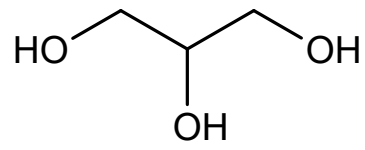
	Lactic acid	LA	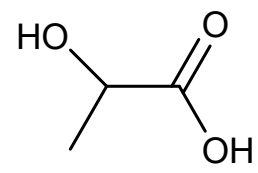
	1,3-Propanediol	PRO	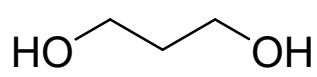
	Urea	URE	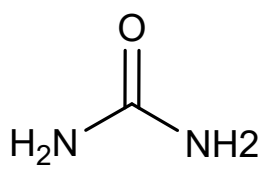

Table S2. Different types of deep eutectic solvents

NO.	HBD	HBA	Mole Ratio	Water
DES1	1,4-butanediol	Choline chloride		
DES2	Citric acid	Choline chloride		
DES3	Glucose	Choline chloride		
DES4	Fructose	Choline chloride		
DES5	Glycerol	Choline chloride		
DES6	Lactic acid	Choline chloride	2:1	12
DES7	1,3-Propanediol	Choline chloride		
DES8	Urea	Choline chloride		
DES9	1,4-butanediol	Betaine		
DES10	Citric acid	Betaine		
DES11	Glucose	Betaine		

DES12	Fructose	Betaine
DES13	Glycerol	Betaine
DES14	Lactic acid	Betaine
DES15	1,3-Propanediol	Betaine
DES16	Urea	Betaine
Water		

Table S3 The design of single-factor experimental levels

Factors	Unit	Levels				
The molar ratio		1:2	1:1	2:1	3:1	4:1
Water content	wt%	30	40	50	60	70
Liquid-material ratio	mL/g	2:1	4:1	6:1	8:1	10:1
Extraction time	h	1.5	2.0	2.5	3.0	3.5

Table S4 Independent variables and levels used for BBD experiments

Variable	Unit	Factor	Level		
			-1	0	1
Water content	wt%	A	40	50	60
Liquid-material ratio	mL/g	B	6:1	8:1	10:1
Extraction time	h	C	2.5	3.0	3.5

Table S5. The σ-profile location of deep eutectic solvents

Deep eutectic solvents	HBD	Non-polar	HBA
DES1	-0.019 e/Å² < σ< 0.0084 e/Å²	-0.0084 e/Å² < σ< 0.0084 e/Å²	0.0084 e/Å² < σ< 0.021 e/Å²
DES2	-0.026 e/Å² < σ< 0.0084 e/Å²		0.0084 e/Å² < σ< 0.020 e/Å²
DES3	-0.021 e/Å² < σ< 0.0084 e/Å²		0.0084 e/Å² < σ< 0.020 e/Å²
DES4	-0.020 e/Å² < σ< 0.0084 e/Å²		0.0084 e/Å² < σ< 0.020 e/Å²
DES5	-0.020 e/Å² < σ< 0.0084 e/Å²		0.0084 e/Å² < σ< 0.021 e/Å²
DES6	-0.023 e/Å² < σ< 0.0084 e/Å²		0.0084 e/Å² < σ< 0.020 e/Å²
DES7	-0.020 e/Å² < σ< 0.0084 e/Å²		0.0084 e/Å² < σ< 0.020 e/Å²
DES8	-0.019 e/Å² < σ< 0.0084 e/Å²		0.0084 e/Å² < σ< 0.021 e/Å²
DES9	-0.019 e/Å² < σ< 0.0084 e/Å²		0.0084 e/Å² < σ< 0.025 e/Å²
DES10	-0.026 e/Å² < σ< 0.0084 e/Å²		0.0084 e/Å² < σ< 0.025 e/Å²
DES11	-0.021 e/Å² < σ< 0.0084 e/Å²		0.0084 e/Å² < σ< 0.025 e/Å²
DES12	-0.021 e/Å² < σ< 0.0084 e/Å²		0.0084 e/Å² < σ< 0.025 e/Å²
DES13	-0.020 e/Å² < σ< 0.0084 e/Å²		0.0084 e/Å² < σ< 0.025 e/Å²

DES14	-0.023 e/Å² < σ< 0.0084 e/Å²	0.0084 e/Å² < σ< 0.025 e/Å²
DES15	-0.020 e/Å² < σ< 0.0084 e/Å²	0.0084 e/Å² < σ< 0.025 e/Å²
DES16	-0.020 e/Å² < σ< 0.0084 e/Å²	0.0084 e/Å² < σ< 0.025 e/Å²
